# Involvement of MdWRKY40 in the defense of mycorrhizal apple against *fusarium solani*

**DOI:** 10.1186/s12870-022-03753-z

**Published:** 2022-08-02

**Authors:** Mei Wang, Weixiao Tang, Li Xiang, Xuesen Chen, Xiang Shen, Chengmiao Yin, Zhiquan Mao

**Affiliations:** 1 State Key Laboratory of Crop Biology / College of Horticultural Science and Engineering, Shandong Agricultural University, Tai’an, 271018 Shandong People’s Republic of China; 2 Forestry College of Shandong Agricultural University, Tai’an, 271018 Shandong China

**Keywords:** Apple, *Fusarium solani*, Biotic stress, Plant-pathogen interaction, Resistance genes

## Abstract

**Background:**

Apple (*Malus domestica* Borkh.) is an important economic crop. The pathological effects of *Fusarium solani*, a species complex of soilborne pathogens, on the root systems of apple plants was unknown. It was unclear how mycorrhizal apple seedlings resist infection by *F. solani*. The transcriptional profiles of mycorrhizal and non-mycorrhizal plants infected by *F. solani* were compared using RNA-Seq.

**Results:**

Infection with *F. solani* significantly reduced the dry weight of apple roots, and the roots of mycorrhizal apple plants were less damaged when the plants were infected with *F. solani*. They also had enhanced activity of antioxidant enzymes and a reduction in the oxidation of membrane lipids. A total of 1839 differentially expressed genes (DEGs) were obtained after mycorrhizal and non-mycorrhizal apple plants were infected with *F. solani*. A gene ontogeny (GO) analysis showed that most of the DEGs were involved in the binding of ADP and calcium ions. In addition, based on a MapMan analysis, a large number of DEGs were found to be involved in the response of mycorrhizal plants to stress. Among them, the overexpressed transcription factor *MdWRKY40* significantly improved the resistance of the apple ‘Orin’ callus to *F. solani* and the expression of the resistance gene *MdGLU* by binding the promoter of *MdGLU*.

**Conclusion:**

This paper outlines how the inoculation of apple seedlings roots by arbuscular mycorrhizal fungi responded to infection with *F. solani* at the transcriptional level. In addition, MdWRKY40 played an important role in the resistance of mycorrhizal apple seedlings to infection with *F. solani*.

**Supplementary Information:**

The online version contains supplementary material available at 10.1186/s12870-022-03753-z.

## Background

Apple is one of the most widely produced and economically important fruit crops in temperate regions [1]. Apple replant disease (ARD) is a phenomenon in which the plant growth is severely inhibited after replanting apple trees at the same site for many years [2]. There are many complex etiologies that cause ARD, and the possible factors vary between different regions or orchards of the same region [3, 4]. The primary cause of ARD is biological factors, because disinfection of the soil can effectively prevent or alleviate the disease [5, 6]. Extensive studies have shown that the major pathogens that lead to ARD are *Cylindrocarpon*, *Fusarium*, *Rhizoctonia*, *Pythium*, and *Phytophthora* [7, 8]. Wang et al. [9] found that there was abundant *Fusarium* spp. in the Bohai Bay, the most abundant species was *Fusarium solani*, and the apple rootstocks are highly sensitive to this pathogen [10, 11]. Members of the *Fusarium solani* species complex (FSSC) are capable of causing disease in many agriculturally important crops [12]. *F. solani* (MG836251.1) is one of the pathogens that has been proven to cause ARD [13]. Infection with *F. solani* depressed the photosystem performance of apple seedling leaves, destroyed the ROS scavenging system, caused oxidative damage, and increased the number of black and brown necrotic spots on apple roots [10, 14]. Those series of physiologic and biochemical reactions can lead to severe root tip necrosis and decay until plant death occurs several days or weeks after *F. solani* infection [15]. Therefore, it is urgent to find an effective method to enhance the resistance of apple rootstocks to *F. solani*.

Arbuscular mycorrhizal fungi (AMF) can infect more than two-thirds of terrestrial plants to form efficient symbiotic relationships on the roots [16]. There is an interaction among AMF, pathogens, and plants [17]. AMF can not only enhance the resistance of the host to environmental stress, such as drought and salt stress, by improving the efficiency of utilization of plant nutrients [18, 19], but also have a significant effect on the induction of plant resistance to soilborne diseases and improve the growth of host plants [20, 21]. Previous studies have shown that AMF effectively provide biological control against soilborne pathogens, such as *F. oxysporum*, *Sclerotium cepivorum* and *Pythium aphanidermatum* [22]. AMF prevent and control soilborne diseases by challenging the growth sites of pathogens in the root systems of host plants and produce wide networks of extraradical mycelia [23, 24]. The AMF-plant symbiotic relationship results in enhanced plant growth, which may be stimulated through several activities, including regulation of the secondary metabolic pathway of the roots of host plants, the increased synthesis and secretion of terpenes and phenolic compounds, promotion of the synthesis of antimicrobial substances, and improvements in the plant defense system [25, 26]. An important number of genes related to hormone signalling are involved in the enhanced resistance or tolerance of mycorrhizal plants to infection by *F. virguliforme* [27]. The systems of mycorrhizal plants play an active role in the disease resistance process, and plants with mycorrhizae are generally more resistant to soilborne pathogens than plants that lack them [28, 29]. The induction of defense responses in mycorrhizal plants was much higher and more quickly than that in non-mycorrhizal plants when infected by pathogens [29]. However, it is unclear how the physiological and molecular responses of mycorrhizal and non-mycorrhizal prevent the infection of apple seedlings with *F. solani*.

In this study, the apple stock M9T337 was used as the experimental material to explore the defense mechanism of mycorrhizal apple seedlings. Several experimental evaluations of the pre-inoculation of AMF were conducted to help improve the resistance of apple root systems to *F. solani*. We hypothesized that the presence of symbiotic apple root systems enhances resistance to *F. solani*. Therefore, the molecular mechanism of the AMF-apple interaction in response to the infection was analyzed by transcriptome. This study provides a basis for understanding the molecular mechanism of mycorrhizal apple defence against infection by *F. solani*.

## Results

### Physiological properties of mycorrhizal apple roots

The plants were harvested 10 days after infection with *F. solani*. No colonization was detected in the non-inoculated AMF seedlings, including NM (non-mycorrhizal plants that had not been inoculated with *F. solani*) and NM-F (non-mycorrhizal plants inoculated with *F. solani*) treatments. In the AMF inoculated plants, AMF and plant roots had established a symbiotic relationship with a colonization rate of 76% in the AM treatment (mycorrhizal plants that had not been inoculated with *F. solani*), but after infection with *F. solani*, the colonization rate was 49% in AM-F treatment (mycorrhizal plants inoculated with *F. solani*). Infection with *F. solani* significantly reduced the dry weight of plants, and the presence of AMF in plants that had been inoculated with *F. solani* significantly reduced the damage of *F. solani* to the plants. The activity of superoxide dismutase (SOD) of the AM was significantly higher than that of the NM treatment, while the activities of other antioxidant enzymes did not differ significantly from the NM treatment. After 5 days of infection with *F. solani*, the SOD and peroxidase (POD) activities of the AM-F treatment increased by 12.5 and 56.4%, respectively, compared with the NM-F treatment, while there was no significant difference in the activity of catalase (CAT) (Fig. 2A). The contents of malondialdehyde (MDA), hydrogen peroxide (H_2_O_2_), and superoxide (O_2_·^−^) in the AMF symbiotic plants were 19.8, 26.1, and 54.2% lower than those in the non-mycorrhizal plants, respectively (Fig. 2B). We also observed a similar trend in the contents of proline (PRO), soluble protein, and soluble sugar (SOG) in the treated plant roots that correlated with the activities of SOD and POD (Fig. 2C).

### DEG screening

To investigate changes in the level of transcription of apple rootstock M9T337 root after inoculation with AMF and *F. solani*, different treatments were assessed. The differentially expressed genes (DEGs) from the four treatments that were enriched and depleted are shown in Fig. 3. A total of 809 DEGs were identified in the AM-F vs. AM treatment, and 996 DEGs were identified in the NM-F vs. NM treatment. A total of 1839 DEGs, the highest number of differential genes, were identified for subsequent differential gene analysis in the AM-F vs. NM-F treatment. The PCA analysis identified a significant separation between the AM-F and NM-F (Fig. S3).

### GO pathway enrichment analysis

In GO enrichment analysis, the genes were annotated and classified by biological processes, cell components and molecular function. In the AM-F vs. NM-F, molecular function was enriched, including ADP binding (GO:0043531), calcium ion binding (GO:0005509), hydrolase activity (GO:0004553), vitamin binding (GO:0019842), polygalacturonase activity (GO:0004650), and thiamine pyrophosphate binding (GO:0004650). The cell periphery (GO:0071944) and cell wall (GO:0005618) were enriched. In addition, the pyridine nucleotide biosynthesis pathway (GO:0019363), carbohydrate catabolic process (GO:0016052), and protein modification (GO:0070647) were enriched (Fig. 4A).

This experiment demonstrated that there are different expression of multiple members of different TF families, including the WRKY, AP2-ERF, bHLH, NAC, MYB, HB, C2H2, and bHLH families (Fig. 4B). There is a class of proteins designated ethylene response factors in TFs that are closely related to this signaling pathway, and the differential expression analysis also verified the relevant data. A large number of genes (MD15G1221100, MD07G1248600, MD05G1311400, MD04G1058200, MD09G1114800, MD15G1055200, MD02G1096500, MD05G1080900, MD10G1094700) were up regulated in ERF family members by infection with *F. solani* after 5 days. Interestingly, the most representative class of TFs were the positive regulatory induction of the WRKY family genes in mycorrhizal plants. *MdWRKY40, MdWRKY41, MdWRKY53, MdWRKY50, MdWRKY24, MdWRKY76, MdWRKY28,* and *MdWRKY6* were significantly up regulated in the AM-F vs. NM-F treatment.

### MapMan analysis

To provide a more comprehensive understanding of the DEGs in interactions of plants and pathogens, we performed a MapMan visual analysis in biotic stress, secondary metabolism (Fig. 5). The genes that encode TIR-NBS-LRR and NB-ARC were up regulated by *F. solani* infected. A large number of genes that are related to hormone signaling, including auxin, brassinolide, abscisic acid, ethylene, jasmonic acid, and salicylic acid; pathogenesis-related proteins, such as PR1 and PR5; and transcription factors, such as WRKY, MYB, ERF and DOF, were enriched (Fig. 5A). *MdMPK1* and *MdMPK18* were up-regulated in the mitogen-activated protein kinase (MAPK) signal pathway.

The biosynthesis of lignin and lignans, flavonoids, phenylpropanoids, and glucosinolates provided the primary secondary metabolic pathways (Fig. 5B). Glucosinolate biosynthesis is thought to produce a chemical barrier against pathogen infection. Nine genes are involved in its for the biosynthetic process, of which only two genes were significantly down regulated. In addition, MD15G1079200, acyl transferase 9 (MD09G1169600), 4-coumarate--CoA ligase-like 5 (MD07G1073800), cinnamoyl-CoA reductase 1 (MD09G1224200), and probable cinnamyl alcohol dehydrogenase 6 (MD15G1008100) in the phenylpropanoid synthesis pathway were significantly upregulated. The solely salicylic acid 3-hydroxylase (MD03G1140400) and hydroquinone glucosyltransferase (MD01G1077200) were up regulated in flavonoid synthesis pathway, seven DEGs were down regulated. Interestingly, DEGs were all up regulated in both the carotenoids and the simple phenolic synthesis pathways.

### Quantitative reverse transcription-PCR (qRT-PCR) validation

Based on the sequencing data from our transcriptional group to validate the RNA-Seq results, 16 upregulated genes and six downregulated genes were verified with qRT-PCR (Fig. 6A). The candidate genes were selected to examine their participation in the process of interaction between plants and pathogens, including a pathogen recognition receptor, signal transduction, transcription factor, and the genes involved in the synthesis of secondary metabolites (Fig. 5). These genes could play an important role in the mycorrhizal plant in response to biological stress reactions by infection with *F. solani*. As shown in Fig. 6B, the trend toward the levels of expression in RNA-Seq and qRT-PCR data was similar, which highlights the reliability of RNA-Seq.

### Overexpression of *MdWRKY40* improves the resistance of ‘Orin’ to *F. solani*

We found the highest relative level of expression of *MdWRKY40* (MD15G1039500) in qRT-PCR validation (Table S3). The open reading frame (ORF) of MdWRKY40 was 912 bp, encoding a 303 amino acid, with one conserved WRKY domain and a zinc-finger motif (Fig. S5). To validate the resistance of *MdWRKY40* to *F. solani*, *MdWRKY40* was chosen to transform the apple callus. Compared with the wild-type (WT) and OE-*MdWRKY40* ‘Orin’ callus that had been infected with *F. solani* after 4 days, the diameter of fungal spots in the OE-*MdWRKY40* was significantly lower than that in the WT, and the diameter of plaque extension in calli was decreased by 56.4% (Fig. 7B,C)*.* To study whether MdWRKY40 regulates the expression of resistance genes, the relative levels of expression of *MdPR1*, *MdPR4*, *MdPR5*, *MdPR8*, *MdECHT*, and *MdGLU* in callus following infection with *F. solani* were measured by qRT-PCR (Fig. 7D). The levels of expression of *MdGLU* in OE-*MdWRKY40* were significantly higher than the levels in wild type (WT) in all the resistance genes tested (*P <* 0.01). This suggests that MdWRKY40 could improve the resistance of callus to *F. solani* by regulating the expression of *MdGLU*.

### MdWRKY40 bound to the *MdGLU* promoter

Previous studies showed that the WRKY transcription factors usually target W-box motifs to activate or inhibit the level of expression of downstream genes [30]. The promoters of *MdGLU* contain three W-boxes (Fig. S5). A yeast one-hybrid test was used to determine whether MdWRKY40 combined with the *MdGLU* promoter. On the media that lacked Trp and His, the optimal concentration to inhibit the expression of HIS3 in the pHIS2 vector was 120 mm 3-amino-1,2,4-triazole (3-AT) for pro*MdECHT*-pHIS2 (Fig. 8B). In the Y1H assays, MdWRKY40 interacted with the promoters of *MdGLU*. When WRKY40 was divided into two fragments (N-terminus and C-terminus), we found that the C-terminus that contains the WRKY domain bound to the *MdGLU* promoter (Fig. 8C). An electrophoretic mobility shift assay (EMSA) confirmed that MdWRKY40 could bind to the second W-box in the *MdGLU* promoter but not to the other three W-boxes (Fig. 8D, Fig. S6). As the concentration of the cold probe increased, the binding weakened, and the addition of the mutant cold probe did not affect the binding. To determine how MdWRKY40 regulates the level of *proMdGLU* activity, luciferase (LUC) reporter assays were performed. MdWRKY40 stimulated the expression of the LUC gene driven by the promoter of *MdGLU* (Fig. 8E). These results indicated that MdWRKY40 activated *proMdGLU.*

## Discussion

### Mycorrhizal symbiosis improved the resistance of apple seedling to *F. solani*

**Fig. 1:**
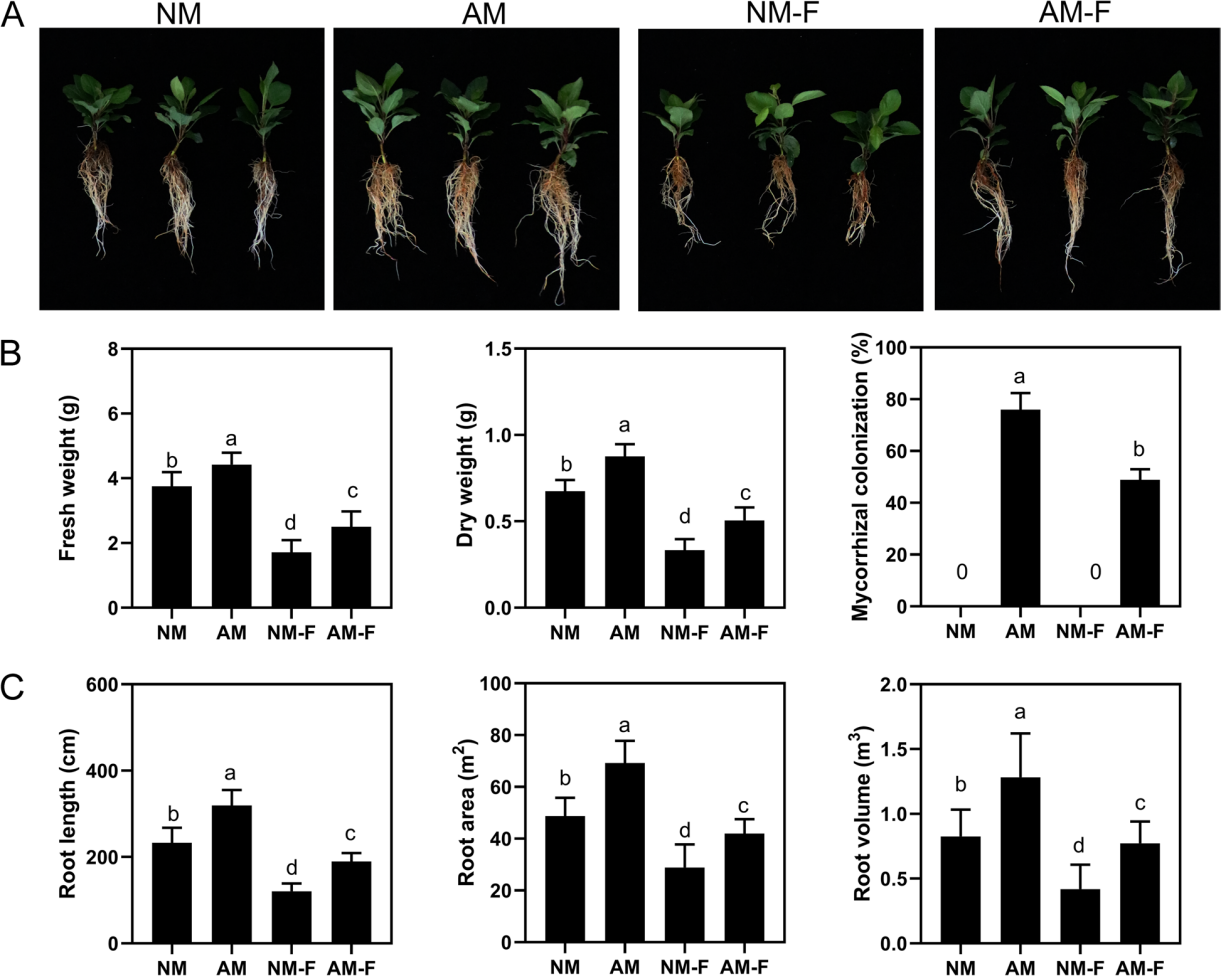
Effects of different treatments on (**A**) plant growth, (**B**) plant biomass and mycorrhizal colonization, (**C**) root morphology of apple seedlings. Different letters represent significant differences at *P* < 0.05

The symbiosis of AMF with plant roots is often mutually beneficial. After establishing a symbiotic system with the host plant roots, the AMF will promote root development and increase the root biomass [31]. A large amount of research has shown that mycorrhizal plants tend to have more developed roots to absorb essential moisture and nutrients for the host plants under adverse conditions [19, 21]. The symbiosis of AMF not only enhances the absorption of soil nutrients by host plant roots but also creates the mycorrhizal pathway for nutrient uptake by the AM fungal mycelia, and plays an important role in the management of abiotic and biotic stress by the plant [32]. In this study, the root length of M9T337 was significantly reduced after infection by *F. solani*. The pre-inoculation of AMF promoted root growth, indicating that after AMF colonized the plant root system, the soilborne pathogens competed with their ecological niche and reduced their rate of invasion on the root epidermis (Fig. 1). Simultaneously, after the formation of mycorrhizal plants, a developed mycorrhizal network formed a frontal physical barrier with the root system, could help the plants to more effectively resist the harm caused by pathogens to plants [33, 34]. AMF inoculated reduce mortalities root changing activities of lignification and increase isozymes against *F. oxysporum* infection. After AMF colonized the plant root system, the lignification degree and the resistance related enzymes activity of mycorrhizal plants roots were increased to resist the infection of *F. oxysporum*, thus reducing the incidence rate [35].

**Fig. 2:**
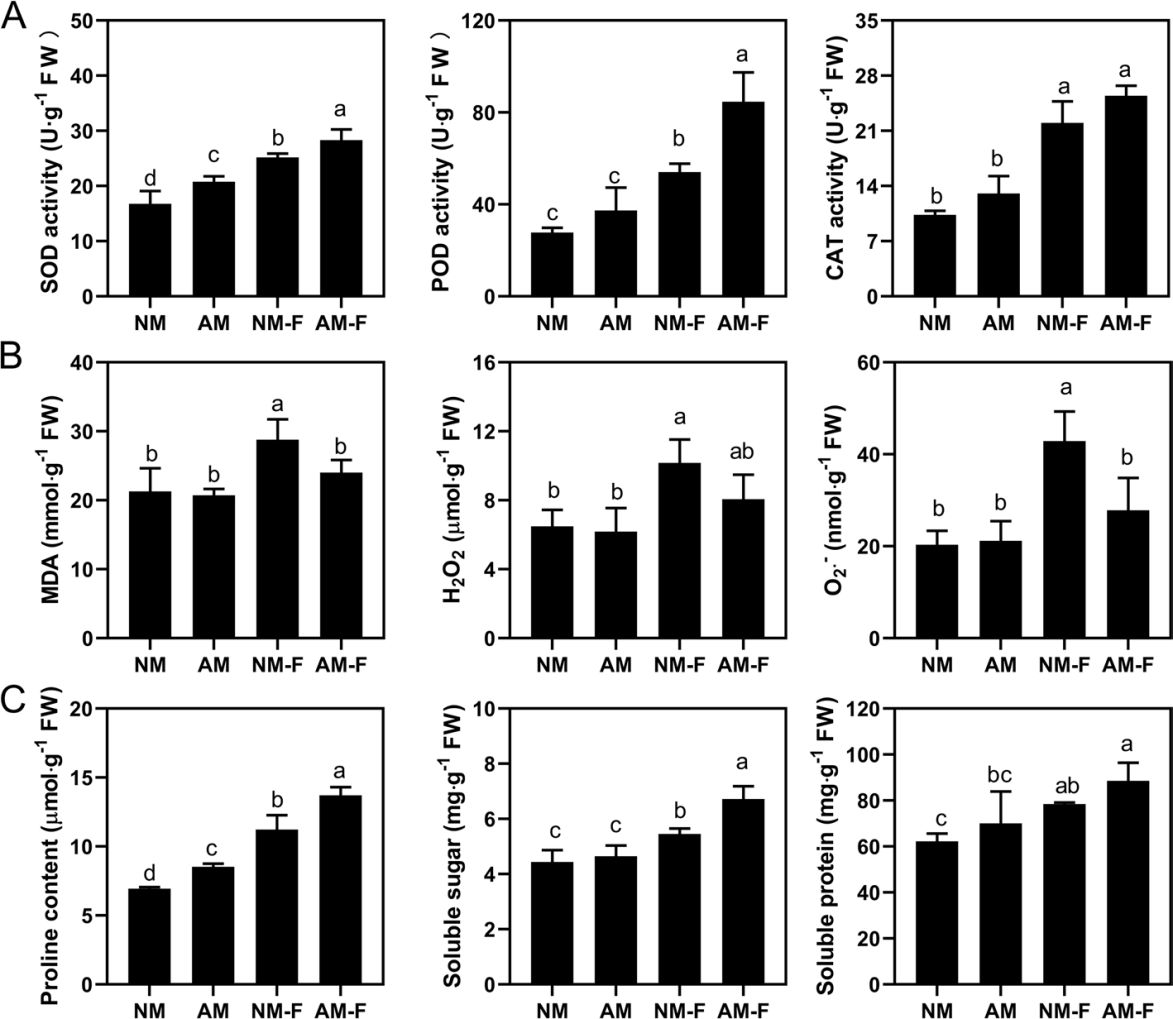
Effects of different treatments on the physiological indicators of apple seedlings. (**A**) defensive enzyme activity, (**B**) reactive oxygen species content, (**C**) osmotic regulator substances. Different letters represent significant differences at *P* < 0.05

*F. solani*, as a plant pathogenic fungus, frequently cause a large amount of necrosis of the roots, resulting in a decline in their photosynthetic ability and other pathological changes [10]. When pathogenic fungi infect plants, the resistance of mycorrhizal plant is often induced during the early stages of infection to reduce the damage of pathogens to plants [28]. Studies have shown that *Funneliformis mosseae*, *Rhizophagus irregularis*, and other AMF have been used to varying degrees to reduce plant diseases caused by pathogenic fungi [36, 37]. Consistent with previous studies, AMF significantly improved the resistance of plants by improving the activities of plant defense enzymes, reducing the oxidation of membrane lipids, and enhancing the plant antioxidant prevention system (Fig. 2). These findings suggest that AMF symbiosis reduced the degree of membrane oxidation and improved the antioxidant activities and permeable regulatory material content to protect the apple root system.

### Mycorrhizal plants respond to *F. solani* infection

**Fig. 3:**
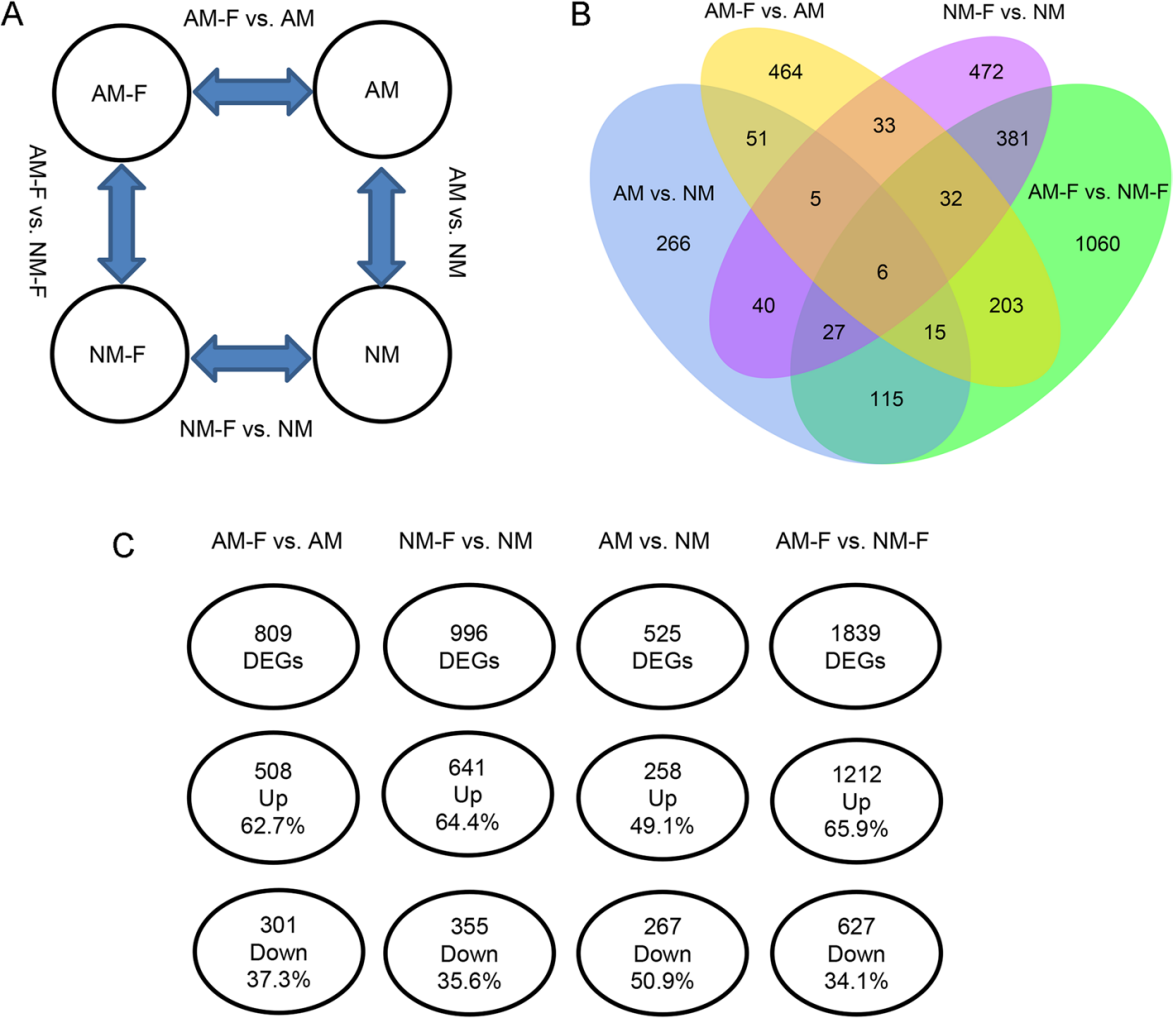
Numbers of differentially expressed genes. (**A**) the experimental design and comparisons, (**B**) Venn diagram, (**C**) Diagram illustrating the number of total, up, and down-regulated genes in the roots of mycorrhizal plants that were infected with *Fusarium solani* (AM-F) or not infected (AM) with *F. solani* and the roots of non-mycorrhizal plants infected with *F. solani* (NM-F) or not infected (NM)

**Fig. 4:**
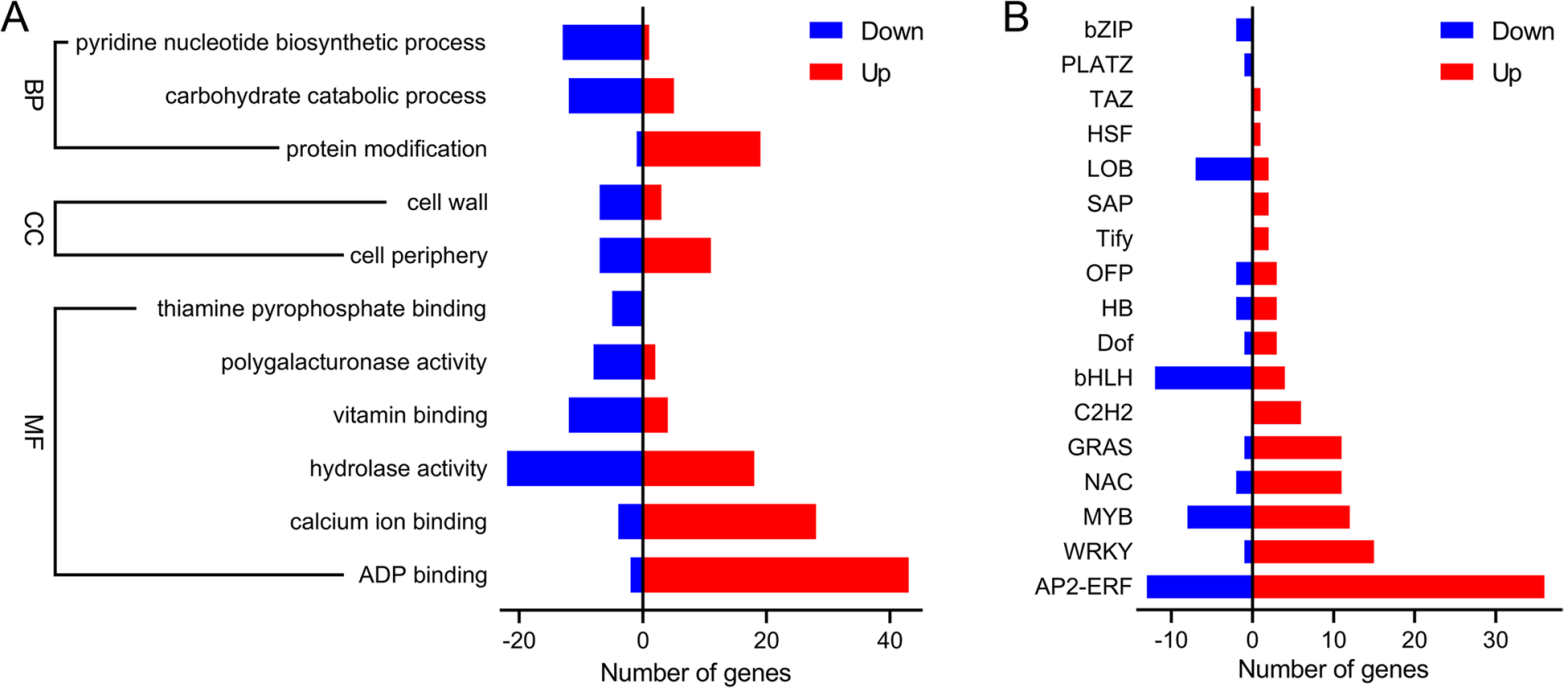
GO enrichment analysis (**A**), and the transcription factor (**B**) numbers of DEGs. The scale bar represents the log2FC (AM-F/NM-F) of DEGs. Red and blue indicate up and down regulated genes, respectively. DEGs, differentially expressed genes; GO, gene ontology

**Fig. 5:**
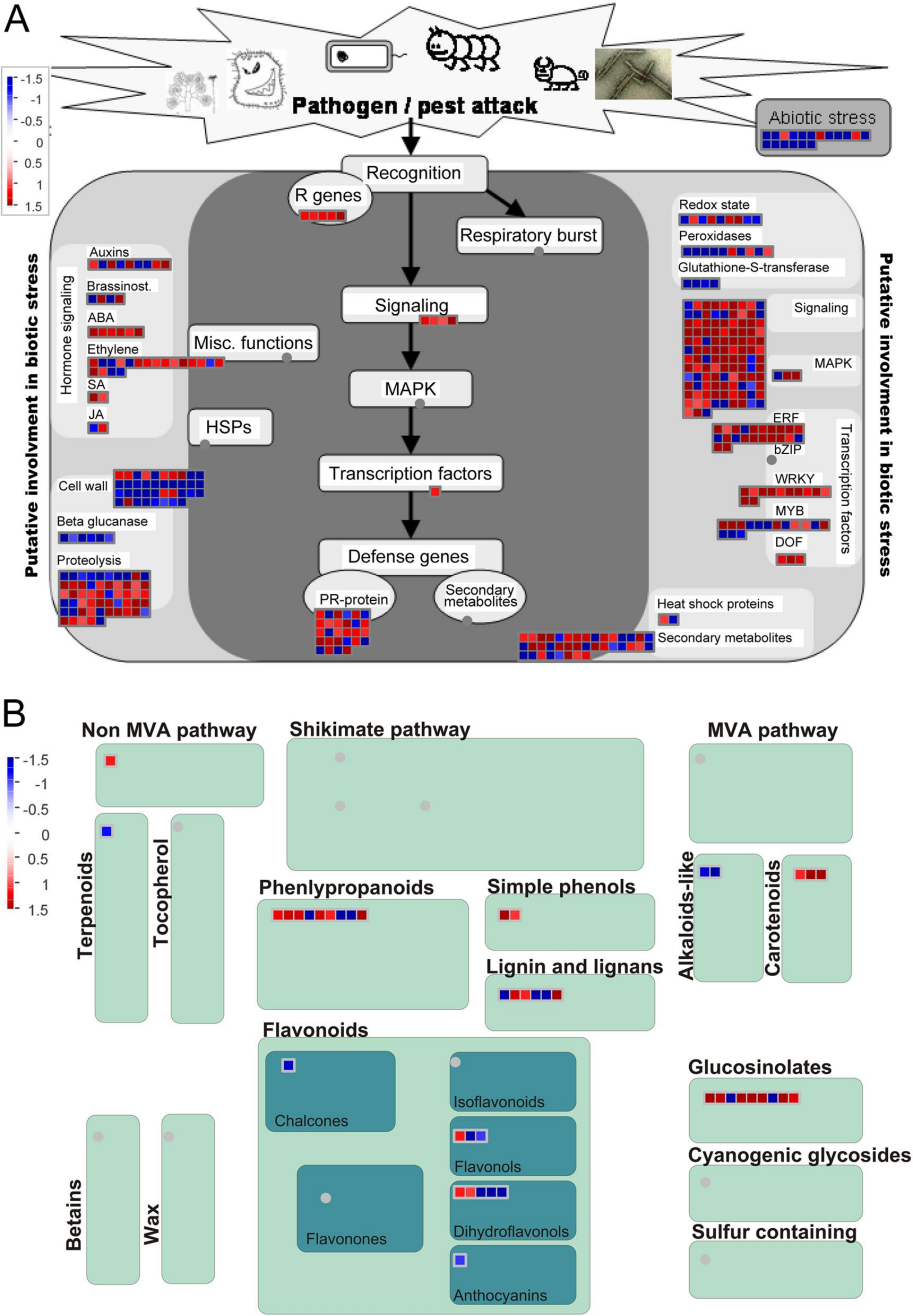
Impact of AMF pre-colonization on gene expression in apple roots infected with *Fusarium solani*. MapMan graphs of (**A**) biotic stress, (**B**) secondary metabolism. The scale bar represents the log2FC (AM-F/NM-F) of the DEGs. Red and blue indicate up and down-regulated genes, respectively

The plant-AMF-pathogen interaction is a complex network system that involves multiple gene expression and complex regulation [34, 38]. After the pathogens invade plants, the plant will increase the expression of resistance genes and promote the transmission of signal substances and plant metabolism to respond quickly to pathogenic infections [39]. Transcriptome data provide the expression of information and the level of expression for genes that are commonly used to identify the differential stresses of functional plants to biological and non-biological factors [40, 41]. To further understand the biological function of the DEGs, we performed GO Pathway significant enrichment and MapMan visualization analyses to determine key differential genes for the main physiological and biochemical metabolic pathways and signal transduction pathways. There were significant morphological differences between the AM and NM roots after pre-inoculation with AMF (Fig. 1). In addition, there were significant differences between gene expression and the DEGs between the AM and NM after infection with *F. solani* (Fig. 3). In the GO enrichment analysis, more DEGs were concentrated in molecular function, with the most upregulated genes found in the ADP binding (GO:0043531) and calcium ion binding (GO:0005509) pathways (Fig. 4A). The shock of calcium ion concentration in the cytoplasm of root epidermal cells is a central signal factor for plant symbiosis with AMF. The calcium-dependent protein kinase activates downstream calcium/calcium-dependent protein kinases to induce the expression of genes related to symbioses [42]. In the presence of pathogens, AMF constantly competes with pathogens to invade sites on the plant root system, and thus, increases the expression of calmodulin protein genes, such as *MdCML37* and *MdCML38* (Fig. 5). The plant cells sense that calmodulin was activated in a relative stress response to protect the cells from a high infiltration environment [43, 44].

During the process of the interactions of plants with pathogens, a series of defense mechanisms were formed, and the metabolic routes described above were closely related to the defense responses of plants. The related genes of apple-AMF symbionts were analyzed using a MapMan analysis to more comprehensively show the changes of genes in related pathways between mycorrhizal and non-mycorrhizal plants by infection with *F. solani*. Cells respond to stress through signaling transduction and the intracellular regulation of plant physiological and biochemical reactions, while the MAPK signaling transduction pathway is in the center of cell signal transduction system [45]. Huang et al. [46] found that the inoculation of AMF resulted in a significant upregulation of *MdMAPK*, along with the functional response of *MdMAPKs* to plant hormone signaling and stress responses under drought stress. Plant hormone signal transduction played an important role in the interactions of mycorrhizae to protect the plants against pathogens [47]. In this study, we identified a significant upregulation of hormone signaling genes and numerous PR genes. The MAPK signaling pathway and the plant hormone signal transduction were essential for the resistance of mycorrhizal plants to pathogens, but the deeper molecular mechanisms still merit further research.

### TFs respond to mycorrhizal plant resistance to *F. solani*

**Fig. 6:**
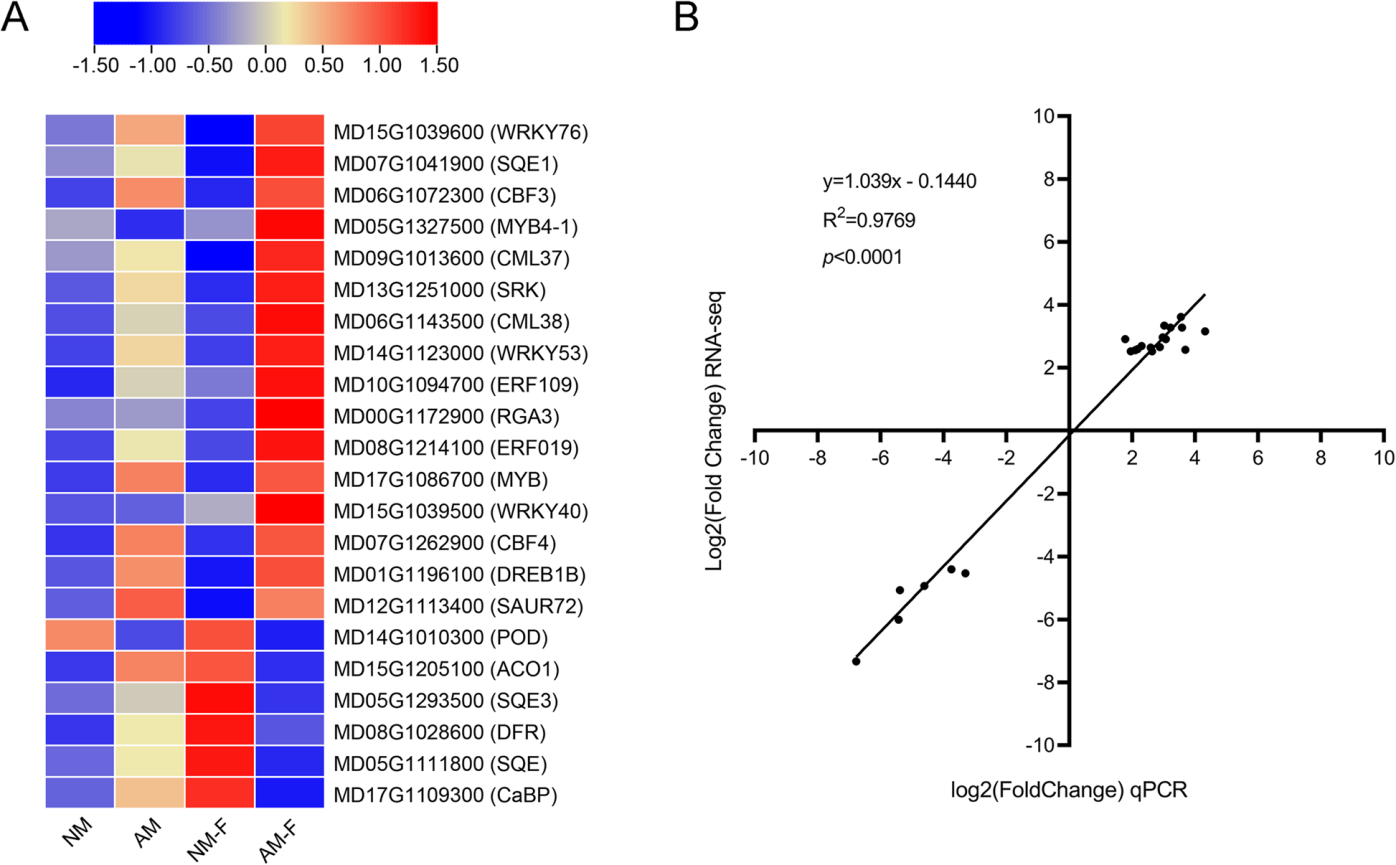
qRT-PCR verification of the genes in biotic stress process. A total of 22 genes were selected for qRT-PCR analysis from the RNA-Seq data. (**A**) A heatmap was generated based on the fold-change values for RT-qPCR and the color scale for values was shown at the top. (**B**) Scatterplots with the correlation between RNA-Seq and qRT-PCR data. qRT-PCR, quantitative reverse transcriptase PCR

TFs play an important role in the process of the plant response to the environment [48, 49]. Extensive studies have shown that the families of transcription factors, such as WRKY, AP2, NAC, MYB, and GRAS, are involved in the regulation of expression of defense-related genes (Fig. 4B). In this study, a large number of AP2, WRKY and MYB transcription factor family genes were involved in the response of mycorrhizal plants against infection with *F. solani*. Only one gene with diminished expression of the WRKY transcription factor was detected in the transcription, and the others increased significantly. The log2 Fold Change of *MdWRKY76*, *MdWRKY53*, and *MdWRKY40* was more than 2.5-fold (Fig. 6). The results of qRT-PCR were consistent with the RNA-Seq data, and *MdWRKY40* was expressed up to 3.69-fold (Table S3).

**Fig. 7:**
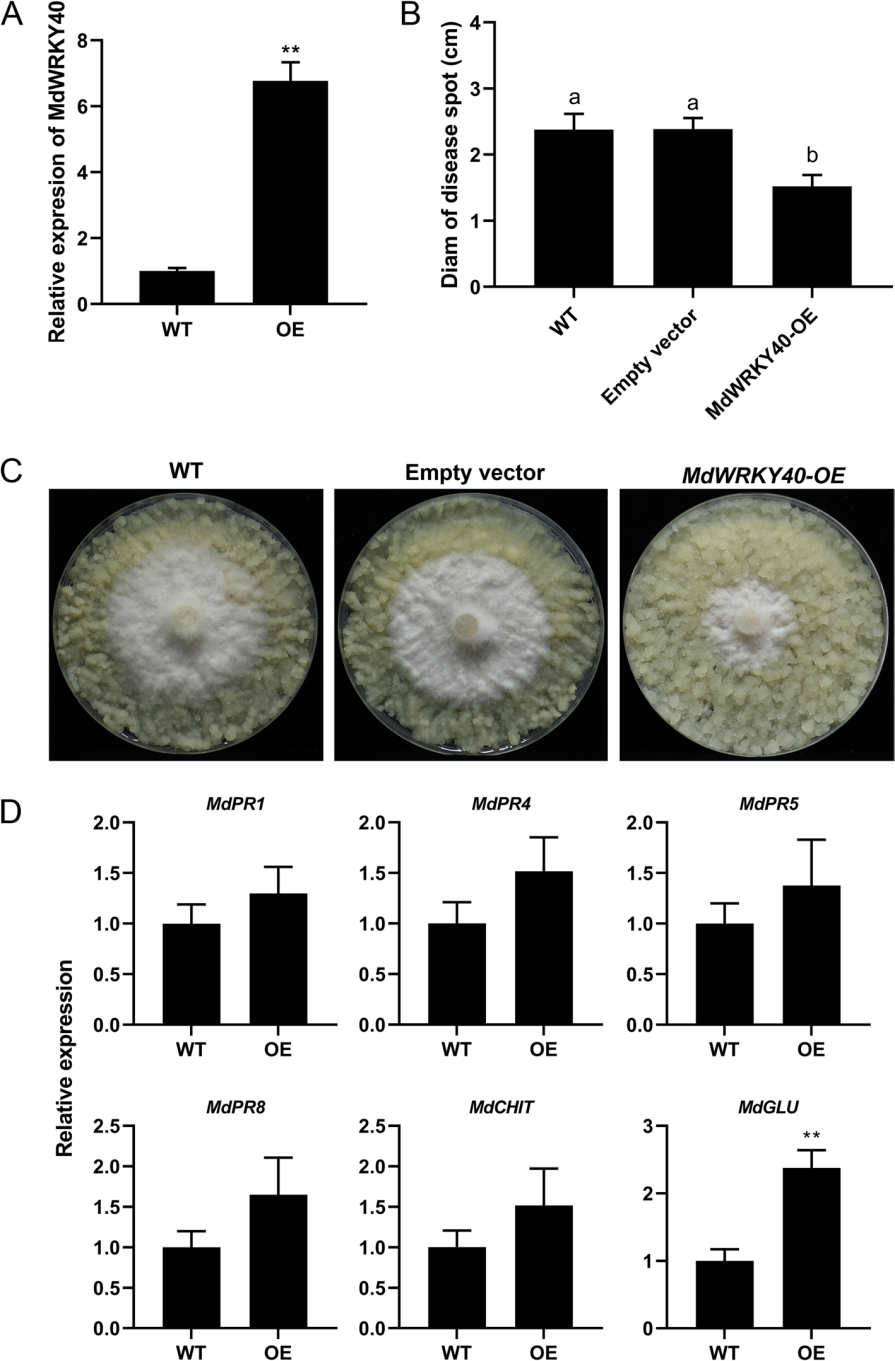
Functional characteristics of ‘Orin’ callus that overexpressed *MdWRKY40*. (**A**) qRT-PCR detection of the expression of *MdWRKY40* in *MdWRKY40*-OE transgenic calli. WT represents wild-type calli. OE represents *MdWRKY40*-OE transgenic lines. (**B**) Diameters of spots in different apple calli after 4 days of infection with *Fusarium solani*. (**C**) Phenotype of WT, empty vector and *MdWRKY40* overexpressing, transgenic apple calli inoculated with *F. solani* after 4 days. (**D**) Transcript levels of *MdPR1*, *MdPR4*, *MdPR5*, *MdPR8*, *MdCHIT* and *MdGLU* in *MdWRKY40*-OE and control calli infected with *F. solani*. OE, overexpression; qRT-PCR, quantitative reverse transcriptase PCR. *P < 0.05. **P < 0.01

**Fig. 8:**
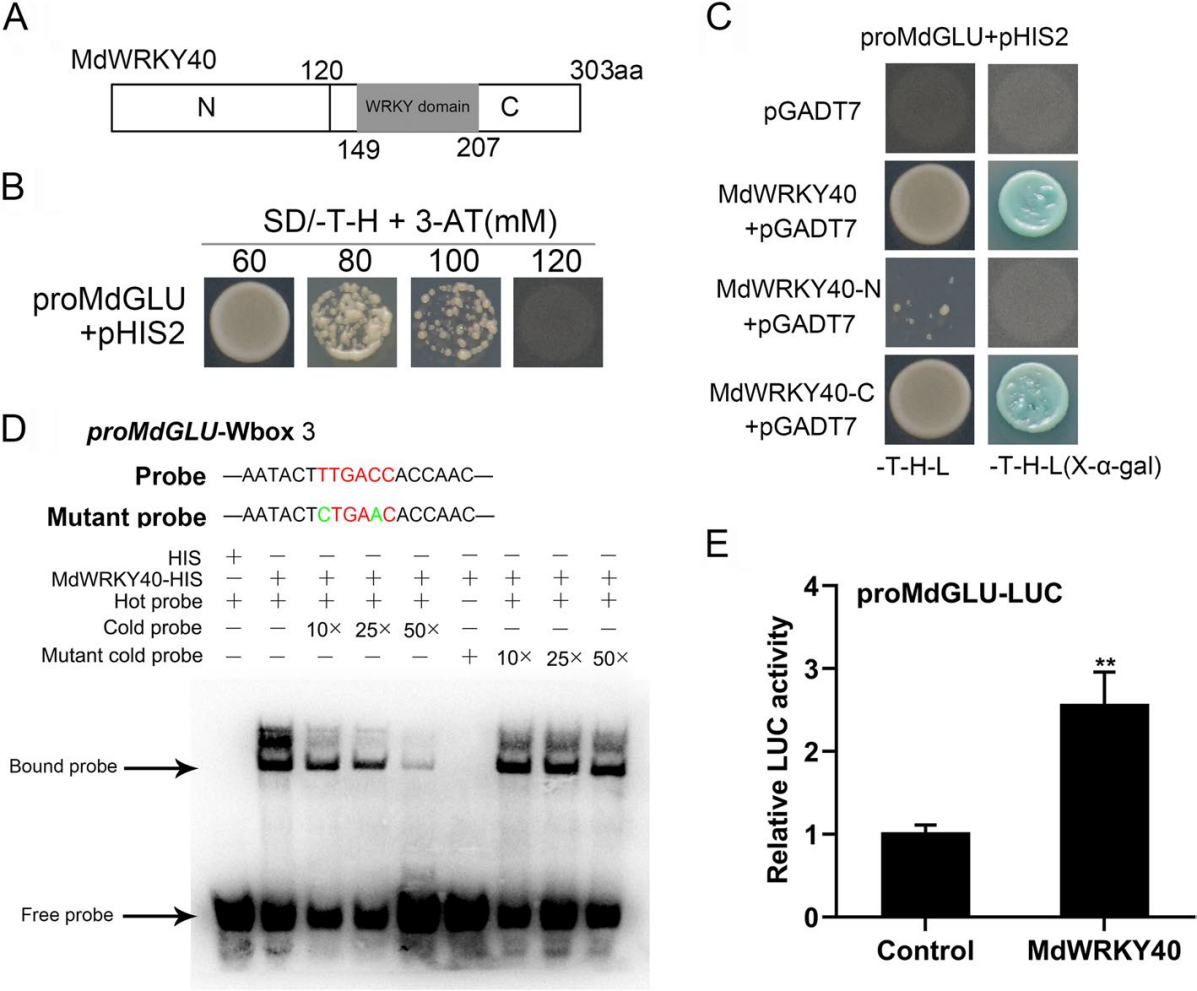
MdWRKY40 binds to the *MdGLU* promoter. (**A**) *MdWRKY40* was divided into two fragments, N (N) terminus, and C (C) terminus. (**B**) The optimal concentration of 3-AT was determined by cloning *proMdGLU* into the pHIS2 vector. (**C**) MdWRKY40 interacted with *MdGLU* promoter fragments as per the Y1H assay. (**D**) An EMSA analysis revealed that MdWRKY40 binds to the W-box II of the *MdGLU* promoter. (**E**) Luciferase reporter (LUC) assays showed the MdWRKY40-mediated activation of *proMdGLU*. EMSA, electrophoretic mobility shift assay. *P < 0.05. **P < 0.01

The overexpression of *VvWRKY18* enhances the resistance of *Arabidopsis thaliana* to *Botrytis cinerea* through activation of the *STILBENE SYNTHASE* (STS) genes [50]. The overexpression of *HbWRKY40* induced resistance to *Colletotrichum gloeosporioides* in tobacco [51]. An evolutionary tree analysis found that MdWRKY40 is a member of WRKY IIa (Fig. S7). We hypothesize that MdWRKY40 played a positive role when mycorrhizal plant roots were infected by *F. solani*. Therefore, the functional validation of *MdWRKY40* was performed (Fig. 7). And through its overexpression in “Orin” calli that the overexpression of *MdWRKY40* enhanced the resistance to *F. solani* and improved the level of transcription of *MdGLU*. YIH, EMSA and LUC report analyses identified the binding of MdWRKY40 to the *MdGLU* promoter, which indicates that it is, involved in the resistance of apple to infection by *F. solani* (Fig. 8).

## Conclusions

The apple seedlings with AMF significantly reduced the deleterious effects of *F. solani* to the root system, improved the antioxidant enzyme activities, and reduced the degree of membrane lipid oxidation. Most of the DEGs involved in plant pathogen interaction, glycolysis/gluconeogenesis, and hormone signal transduction pathway were identified through GO analyses. The mycorrhizal and non-mycorrhizal apple plants were subjected to oxidative stress after inoculation with *F. solani*. However, compared with non-mycorrhizal plants, an enormous number of resistance genes were involved in the stress response process to reduce the amount of oxidative damage. MdWRKY40 improved its expression by binding with the *MdGLU* promoter to participate in mycorrhizal apple seedlings against the infection of *F. solani*.

## Methods

### Plant growth and plant infection

Apple stock M9T337 was grown in an illuminated greenhouse at 20–30 °C (daytime) and 0–15 °C (night) with a relative humidity of 55–65%. The apple stock M9T337 was obtained from Shandong Horticultural Techniques Services Co., Ltd., Tai’an, Shandong, China. The ‘Orin’ apple callus was provided by Prof. Xue-sen Chen of the State Key Laboratory of Crop Biology (Tai’an, China). The appropriate permission was obtained for the plant collection and it’s use was executed in accordance with relevant guidelines. ‘Orin’ apple calli (*Malus domestica* cv. ‘Orin’) were cultured in subculture medium that contained MS, 2.5 mg·L^− 1^ 2,4-D, and 0.5 mg·L^− 1^ 6-indole-3-butyric acid at room temperature in the dark, and the subculture medium was renewed every 15 days for genetic transformation. *F. solani* (MG836251.1) was isolated from the roots of a replanted apple tree (Fig. S1) [52]. The hyphae of *F. solani* were inoculated in sterilized Potato Dextrose Broth (PDB) media and cultured in a shaker at 28 °C for one week. The hyphae were filtered with sterile gauze, and the number of plates was counted. The spores were adjusted to 10^5^·mL^− 1^ with sterile water. Arbuscular mycorrhizal fungi used in this study was *Paraglomus* sp. SW1 (CGMCCNO. 20,744), which was provided by Shandong Agricultural University (Tai’an, China). The inoculant was a mixture of vermiculite, spores (spore density 28·g^− 1^), hyphae and colonized root segments. The systems of mycorrhizal plant roots were grown for 4 weeks using *Paraglomus* sp. SW1.

We established four treatments: (1) NM: non-mycorrhizal plants that had not been inoculated with *F. solani*; (2) AM: mycorrhizal plants that had not been inoculated with *F. solani*; (3) NM-F: non-mycorrhizal plants inoculated with *F. solani*; and (4) AM-F: mycorrhizal plants inoculated with *F. solani*. After the apple stock M9T337 plantlets grew for 4 weeks with 1% AMF inoculum, 50 mL of a solution of *F. solani* spores was used to treat them. After 5 days, the apple roots were collected for transcriptome sequencing, qRT-PCR verification, and an analysis of the enzymes involved in resistance. The apple seedlings were randomly collected after 10 days post-infection to determine the apple biomass, root morphology and mycorrhizal colonization rate.

### Rate of mycorrhizal colonization

The rate of mycorrhizal colonization was determined as described by Giovannetti and Mosse [53]. A 1 cm root segment was digested with a solution of 10% KOH at 90 °C for 20 min, acidified with 2% HCl for 5 min, stained with 0.05% triphenyl blue lactic acid glycerin solution (lactic acid/glycerin =1/1) at 90 °C for 30 min, and decolorized overnight with a solution of lactic acid glycerin (lactic acid/glycerin/water = 1/1/1 [v/v/v]). The root segments were observed under a microscope. The rate of infection of each root segment was assessed by the number of root mycorrhizal structures in each section and expressed as 0, 10, 20, ..., 100% of the root. The mycorrhizal colonization rate (%) was calculated as follows:

$$\mathrm{Mycorrhizal}\ \mathrm{colonization}\ \mathrm{rate}=\Sigma\ \left(0\times \mathrm{root}\ \mathrm{segment}\ \mathrm{number}+10\%\times \mathrm{root}\ \mathrm{segment}\ \mathrm{number}+20\%\times \mathrm{root}\ \mathrm{segment}\ \mathrm{number}+\dots \dots +100\%\times \mathrm{root}\ \mathrm{segment}\ \mathrm{number}\right)/\mathrm{Total}\ \mathrm{root}\ \mathrm{segment}\ \mathrm{number}.$$

### RNA extraction, cDNA library construction, and Illumina sequencing

Total RNA was isolated using a RNA Prep Pure Plant Kit (CWBIO, Beijing, China) according to the manufacturer’s instructions. Total RNA was tested with an Agilent 2100 Bioanalyzer (Agilent Technologies, Santa Clara, CA, USA). The synthesis of cDNA was performed using HiScript II 1st Strand cDNA Synthesis Kit (Vazyme, Nanjing, China). cDNA fragments that were preferentially 250 ~ 300 bp long were purified with an AMPure XP system (Beckman Coulter, Brea, CA, USA). The PCR products were purified again after PCR amplification, and the cDNA library was finally obtained [54]. And cDNA library was assessed on an Agilent Bioanalyzer 2100 system (Agilent Technologies). The libraries were sequenced on a HiSeq® X Ten System (Illumina, San Diego, CA) by Novogene Co., Ltd. (Beijing, China).

### Transcriptomic data analysis

To ensure the quality and reliability of the data analysis, it is necessary to filter the original data, including the removal of reads with an adapter, and remove the reads that contain N. N indicates that the nucleobase information cannot be determined. The low quality reads were then removed. The base number of qPHRED was ≤20, which comprised ≥50% of the whole read length. Moreover, the contents of Q20, Q30, and GC in the clean data were calculated. All follow-up analyses were based on the clean data high quality. Clean data obtained by filtering the raw data were aligned to the reference genome sequence of *Malus* × *domestica* (GDDH13 v. 1.1) by HISAT2 (v. 2.0.5).

The program FeatureCounts (v. 1.5.0-p3) was used to calculate the readings that mapped to each gene [55]. The FPKM of each gene was calculated based on the length of gene, and the reading that mapped to the gene was calculated. DESeq2 software (1.20.0) was used to analyze the differential expression between the four treatments [56]. DEGs with |log2(Fold Change)| ≥ 1 and *P*-value < 0.05 were considered as differentially expressed genes (DEGs) after multiple correction for subsequent analyses. The clusterProfiler R package (3.4.4) was used for the Gene Ontology (GO) enrichment analysis of DEGs [57].

MapMan software (version 3.6.0RC1) (http://mapman.gabipd.org/web/guest/mapman) was used to generate functional assignments in the different pathways [58] for each input gene and the data visualization/interpretation of apple gene expression. Gene annotation in the pathway analysis was prepared via Mercator online software within the PlabiPD website (https://www.plabipd.de/portal/mercator4) based on the DNA sequence and followed the default annotation parameter.

### Measurement of root physiological parameters

The root physiological parameters included the activities of SOD, CAT, and POD, the contents of PRO, SUG, MDA, H_2_O_2_ and O_2_·^−^. Each physiological parameter was measured using kits obtained from Suzhou Keming Biotechnology Co., Ltd. (Suzhou, China). All the measurements were performed three times, and the average was calculated for further analysis.

### Gene cloning and function validation of *MdWRKY40*

*MdWRKY40* (MD15G1039500) sequence information was obtained from the apple genome database (http://www.rosaceae.org). Total RNA extraction and cDNA synthesis were conducted as described by RNA Extraction, cDNA library construction, and Illumina sequencing section. A fragment of 912 bp was obtained by PCR amplification, cloned into the pLB vector (TianGen, http://www.tiangen.com/).

The CDS of MdWRKY40 was inserted into the PRI101-AN vector, which has a green fluorescent protein (GFP) tag. The constructed overexpression vector was transformed into *Agrobacterium tumefaciens* LBA4404. The positive *A. tumefaciens* was obtained by PCR and used to infect ‘Orin’ callus. The infected calli were cultured on MS solid media at 24 °C in the dark for 1–2 days. It was then transferred to a screening medium that contained 50 mg·L^− 1^ kanamycin and 250 mg·L^− 1^ carbenicillin. The overexpression of *MdWRKY40* was confirmed by PCR.

### Yeast one-hybrid test

The target gene fragments *MdWRKY40*, *MdWRKY40*-N (0–120 aa), *MdWRKY40*-C (121–303 aa) were ligated into the pGADT7 vector. The fragment of *MdGLU* promoter was cloned into the pHIS2 vector. A yeast one-hybrid test was conducted according to the manufacturer’s instructions of Yeast transformation system 2 (TaKaRa, Dalian, China). The yeast one-hybrid strain was Y187 (Clontech, Takara Bio USA, San Jose, CA, USA). The yeast strain Y187 that contained the recombinant pHIS2 vectors was grown on –Trp/−His (−T/−H) screening media with different 3-AT concentrations to determine the optimal concentrations. To determine the interactions, the Y187 yeast strain that harbored the recombinant pGADT7 and pHIS2 plasmids was spotted onto media that lacked Trp, His, and Leu. The control was an empty pGADT7 plasmid. The primers used are listed in Supplemental Table S2.

### Electrophoretic mobility shift assays

The sequence of *MdWRKY40* was inserted into the pET-32a (+) expression vector (Novagen, Madison, WI, USA). The recombinant MdWRKY40 protein was expressed in *E. coli* BL21 (DE3), and the fusion protein MdWRKY40-His was purified by a His-Tagged Protein Purification Kit (CWBIO, Beijing, China). The biotinylated probe was synthesized by Sangon Biotech Co., Ltd. (Shanghai, China). The fusion protein, probe, and binding buffer were mixed in a centrifuge tube and incubated at 24 °C for 30 min. After 50% glycerol and 5 × loading buffer were added to the sample, non-denaturing acrylamide gel electrophoresis was performed, and the protein-nucleic acid strip was transferred to film placed on nylon. After the completion of UV crosslinking, the preheated Blocking Buffer closure was added, then HRP Conjugate and 20 mL new Blocking Buffer (ThermoFisher, Shanghai, China) were incubated at room temperature for 15 min. The Washing Buffer (ThermoFisher) was used after washing and developing.

### LUC activity

*MdWRKY40* full length CDS inserted into the pHBT-AvrRpm 1 carrier and promoter segments of *MdGLU* into the pFRK1-LUC-nos carrier. Both plasmids were converted simultaneously from the protoplasm of apple callus and then expressed for 6 h at 24 °C. Subsequently, the protoplasm was suspended in 100 μL of cell lysate. The 5 μL cell extract and 20 μL 1 mmol·L^− 1^ 4-MUG were incubated at 37 °C at 1 h, and the 100 μL of 0.2 mol·L^− 1^ sodium acetate was added to the termination reaction. The LUC activity was determined using the Luciferase Reporting Analysis System (Promega, Madison, WI, USA).

### Quantitative reverse transcription-PCR (qRT-PCR)

The cDNA was synthesized using a HiScript II 1st Strand cDNA Synthesis Kit (Vazyme, Nanjing, China). The reverse transcription reactions began with 500 ng of total RNA. The resulting first stand cDNA was diluted 10-fold with ddH_2_O, and then used as templates for qRT-PCR assays. The primers were designed for qRT-PCR by Primer 6.0 software (Premier Biosoft, Palo Alto, CA, USA). The internal reference gene was actin. The reaction system in the PCR of each primer was SYBR Green Mix 5 μL, primer (10 μM) 0.3 μL, cDNA 1 μL, and dd H_2_O 3.4 μL. The PCR procedures were 50 °C 2 min, 95 °C 10 min, 95 °C 15 s, 65 °C 60 s, 72 °C (30 cycles), and 72 °C 10 min. The primers were synthesized by Sangon Biotech Co., Ltd. The relative quantitative method used the 2^-∆∆CT^ method [59]. Each sample had three biological replicates. The primer sequences are shown in Supplementary Table S2.

### Data analysis

The experimental data was expressed as the means and standard deviation (SD) of three biological replicates. The plant growth, mycorrhizal colonization rate and physiological data were analyzed by Duncan’s test at the 0.05 level using SPSS v. 19.0 (IBM, Inc., Armonk, NY, USA). The qRT-PCR data were analyzed by t-test. An asterisk (*) indicates significant differences, where ‘*’ represents significant differences at *P* < 0.05, and ‘**’ represents highly significant differences at *P* < 0.01. The graphs were constructed using GraphPad Prism 8 (San Diego, CA, USA). The annotations of the DEGs were based on the databases of GO and MapMan software.

## Supplementary Information

**Additional file 1: Supplementary Table S1.** Sequencing data quality statistics.

**Additional file 2: Supplementary Table S2.** List of primers used in this research.

**Additional file 3: Supplementary Table S3.** FPKM values and qRT-PCR data of 22 candidate DEGs. DEGs, differentially expressed genes; RPKM, fragments per kilobase of transcript per million mapped fragments; qRT-PCR, quantitative reverse transcriptase-PCR.

**Additional file 4: Supplementary Fig. S1.** The morphological observation of *F. solani*.

**Additional file 5: Supplementary Fig. S2.** The development of *Paraglomus* sp. SW1 in M9T337 seedling roots.

**Additional file 6: Supplementary Fig. S3.** Principal Coordinate Analysis of the transcriptome.

**Additional file 7: Supplementary Fig. S4.** MdWRKY40 and MD15G1039500 sequence alignment.

**Additional file 8: Supplementary Fig. S5.** Analysis of the MdGLU promotor sequence.

**Additional file 9: Supplementary Fig. S6.** Electrophoretic mobility shift assay (EMSA) showing the binding of MdWRKY40 to the W-box motif in the promoters of *MdGLU*.

**Additional file 10: Supplementary Fig. S7.** Phylogenetic tree constructed from protein sequences for WRKY transcription factors. The *Arabidopsis thaliana* WRKYs were obtained from the TAIR database (https://www.arabidopsis.org/).

## Data Availability

The raw sequence data are available in the NCBI Sequence Read Archive (SRA) repository. The accession number is PRJNA752816, and SRA RunSelector as follows: https://www.ncbi.nlm.nih.gov/Traces/study/?acc=PRJNA752816. All data supporting the conclusions of this article are included in the article and its additional files.

## Abbreviations

RNA-seq: RNA sequencing
DEGs: Differentially expressed genes
GO: Gene ontogeny
MAPK: Mitogen-activated protein kinase
ARD: Apple replant disease
AMF: Arbuscular mycorrhizal fungi
AM: Mycorrhizal plants that had not been inoculated with *F. solani*
AM-F: Mycorrhizal plants inoculated with *F. solani*
NM: Non-mycorrhizal plants that had not been inoculated with *F. solani*
NM-F: Non-mycorrhizal plants inoculated with *F. solani*
SOD: Superoxide dismutase
POD: Peroxidase
CAT: Catalase
MDA: Malondialdehyde
PRO: Proline
SOG: Soluble sugar
PCA: Principal components analysis
SA: Salicylic acid
JA: Jasmonate acid
AOS: Allene oxide synthase
TFs: Transcription factors
ETI: Effector-Triggered immunity
qRT‑PCR: Quantitative reverse transcriptase-PCR
WT: Wild-type
OE: Over-expression
LUC: Luciferase
CML: Calmodulin-like protein
EMSA: Electrophoretic mobility shift assay

## References

[1] Duan NB, Yang B, Sun H, Wang N, Ma YM, Li MJ (2017). Genome re-sequencing reveals the history of apple and supports a two-stage model for fruit enlargement. Nat Commun.

[2] Sharma N, Verma P, Singh DN (2020). Causes and control measures of apple replant problem. IJBSM..

[3] Yin CM, Wang M, Wang JY, Chen XS, Shen X, Zhang M (2017). The research advance on apple replant disease. Acta Hort Sin.

[4] Tewoldemedhin YT, Mazzola M, Labuschagne I, McLeod A. () A multi-phasic approach reveals that apple replant disease is caused by multiple biological agents, with some agents acting synergistically. 2011;43:1917–27. 10.1016/j.soilbio.2011.05.014.

[5] Sewell G, Roberts AL, Elsey RF (1992). Apple replant disease: the assessment and results of seedling bio-assays of growth responses to soil fumigation with chloropicrin. Ann Appl Biol.

[6] van Schoor L, Denman S, Cook N (2009). Characterisation of apple replant disease under south African conditions and potential biological management strategies. Sci Hortic.

[7] Tewoldemedhin YT, Mazzola M, Botha WJ, Spies CF, McLeod A (2011). Characterization of fungi (*fusarium* and *Rhizoctonia*) and oomycetes (*Phytophthora* and *Pythium*) associated with apple orchards in South Africa. Eur J Plant Pathol.

[8] Strauss S, Kluepfel D (2015). Anaerobic soil disinfestation: a chemical-independent approach to preplant control of plant pathogens. J. Integr. Agr..

[9] Wang G, Yin C, Pan FB, Wang XB, Xiang L, Wang YF (2018). Analysis of the fungal community in apple replanted soil around Bohai gulf. Hortic Plant J.

[10] Yan K, Han G, Ren C, Zhao S, Wu X, Bian T (2018). *Fusarium solani* infection depressed photosystem performance by inducing foliage wilting in apple seedlings. Front Plant Sci.

[11] Xiang L, Wang M, Pan FB, Wang GS, Jiang WT, Wang YF (2021). Transcriptome analysis malus domestica ‘M9T337’ root molecular responses to *fusarium solani* infection. Physiol Mol Plant P.

[12] Coleman JJ (2016). The *fusarium solani* species complex: ubiquitous pathogens of agricultural importance. Mol Plant Pathol.

[13] Xiang L, Wang M, Huang JX, Jiang WT, Yan ZB, Chen XS (2022). MdWRKY74 is involved in resistance response to apple replant disease. Plant Growth Regul.

[14] Xiang L, Zhao L, Wang M, Huang J, Chen X, Yin C (2021). Physiological responses of apple rootstock M.9 to infection by *fusarium solani*. Hortsci..

[15] Xu SS, Kan W, Kong BH, Ma J, Yang GZ (2019). First report of *fusarium oxysporum* and *fusarium solani* causing root rot on *Malania oleifera* in China. Plant Dis.

[16] Zhang L, Zhou J, George TS, Limpens E, Feng G (2021). Arbuscular mycorrhizal fungi conducting the hyphosphere bacterial orchestra. Trends Plant Sci.

[17] Aguilar R, Carreón-Abud Y, López-Carmona D, Larsen J (2017). Organic fertilizers alter the composition of pathogens and arbuscular mycorrhizal fungi in maize roots. J Phytopathol.

[18] Gu LJ, Zhao ML, Ge M, Zhu SW, Cheng BJ, Li XY. Transcriptome analysis reveals comprehensive responses to cadmium stressin maize inoculated with arbuscular mycorrhizal fungi. 2019;186:109744. 10.1016/j.ecoenv.2019.109744. 10.1016/j.ecoenv.2019.109744 31627093

[19] Latef AAHA, Hashem A, Rasool S, Abd_Allah EF, Alqarawi AA, Egamberdieva D, et al. Arbuscular mycorrhizal symbiosis and abiotic stress in plants: a review. J Plant Biol 2016;59(5):407–426. 10.1007/s12374-016-0237-7.

[20] Chen Q, Wu WW, Qi SS, Cheng H, Li Q, Ran Q (2021). Arbuscular mycorrhizal fungi improve the growth and disease resistance of the invasive plant *Wedelia trilobata*. J Appl Microbiol.

[21] de Novais CB, Sbrana C, da Conceição JE, Rouws LFM, Giovannetti M (2020). Mycorrhizal networks facilitate the colonization of legume roots by a symbiotic nitrogen-fixing bacterium. Mycorrhiza..

[22] Hou SW, Hu JL, Wu FY, Lin XG (2018). The disease suppression and the related application of arbuscular mycorrhizal fungi. China J Appl Environ Biol.

[23] Vigo C, Norman JR, Hooker JE (2001). Biocontrol of the pathogen *Phytophthora parasitica* by arbuscular mycorrhizal fungi is a consequence of effects on infection loci. Plant Pathol.

[24] Pepe A, Giovannetti M, Sbrana C (2018). Lifespan and functionality of mycorrhizal fungal mycelium are uncoupled from host plant lifespan. Sci Rep.

[25] da Trindade R, Almeida L, Xavier L, Lins AL, Andrade EH, Maia JG (2019). Arbuscular mycorrhizal fungi colonization promotes changes in the volatile compounds and enzymatic activity of lipoxygenase and phenylalanine ammonia lyase in *Piper nigrum* L. ‘Bragantina’. Plants..

[26] Bagy HMMK, Hassan EA, Nafady NA, Dawood MFA (2019). Efficacy of arbuscular mycorrhizal fungi and endophytic strain *Epicoccum nigrum* ASU11 as biocontrol agents against blackleg disease of potato caused by bacterial strain *Pectobacterium carotovora* subsp. *atrosepticum* PHY7. Biol Control.

[27] Marquez N, Giachero M, Gallou A (2019). Transcriptome analysis of mycorrhizal and nonmycorrhizal soybean plantlets upon infection with *fusarium virguliforme*, one causal agent of sudden death syndrome. Plant Pathol.

[28] Cui L, Guo F, Zhang JL, Yang S, Meng JJ, Geng Y (2019). Arbuscular mycorrhizal fungi combined with exogenous calcium improves the growth of peanut (*Arachis hypogaea* L.) seedlings under continuous cropping. J Integr Agr.

[29] Song YY, Chen DM, Lu K, Sun ZX, Zeng RS (2015). Enhanced tomato disease resistance primed by arbuscular mycorrhizal fungus. Front Plant Sci.

[30] Zhao XY, Qi CH, Jiang H, Zhong MS, You CX, Li YY (2019). MdHIR4 transcription and translation levels associated with disease in apple are regulated by MdWRKY31. Plant Mol Biol.

[31] Ma J, Martina J, Li Y, Yu X, He C (2015). Impact of arbuscular mycorrhizal fungi (AMF) on cucumber growth and phosphorus uptake under cold stress. Funct Plant Biol.

[32] Li N, Wang C, Li X, Liu M (2019). Effects of earthworms and arbuscular mycorrhizal fungi on preventing *fusarium oxysporum* infection in the strawberry plant. Plant Soil.

[33] Meng L, Zhang A, Wang F, Han X, Wang D, Li S (2015). Arbuscular mycorrhizal fungi and rhizobium facilitate nitrogen uptake and transfer in soybean/maize intercropping system. Front Plant Sci.

[34] Hou S, Hu J, Wu F, Lin X (2018). The function and potential application of disease suppression by arbuscular mycorrhizal fungi. Chin J Appl Environ Biol.

[35] Jalaluldeen AM, Sijam K, Ramadan NA (2020). Active changes of lignifications-related enzymes in chili pepper response to *Glomus mosseae* against *fusarium oxysporum*. Aust J Basic Appl Sci.

[36] Ravnskov S, Cabral C, Larsen J (2019). Mycorrhiza induced tolerance in Cucumis sativus against root rot caused by *Pythium ultimum* depends on fungal species in the arbuscular mycorrhizal symbiosis. Biol Control.

[37] Trindade R, Almeida L, Xavier L, Andrade EH, Silva J (2021). Influence on secondary metabolism of *Piper nigrum* L. by co-inoculation with arbuscular mycorrhizal fungi and *fusarium solani* f. sp. *piperis*. Microorganisms..

[38] Wang X, Ding T, Li Y, Guo Y, Li Y, Duan T. Dual inoculation of alfalfa (*Medicago sativa* L.) with *Funnelliformis mosseae* and *Sinorhizobium medicae* can reduce *fusarium* wilt. J Appl Microbiol. 2020;129. 10.1111/jam.14645. 10.1111/jam.14645 32215998

[39] Zhang WZ, Gu LJ, Duan TY (2018). Research progress on the mechanism of AM fungi for improving plant stress resistance. Pratacultural Sci.

[40] Yu XM, Wang JZ, Xue XM, Chen R, Nie PX, Wang GP, Han XP (2020). Preliminary studies on the mechanism of bitter pit in apple based on whole-transcriptomic sequencing analysis. Acta Phytopathol Sin.

[41] Tian L, Chang C, Ma L, Nasir F, Tian C (2019). Comparative study of the mycorrhizal root transcriptomes of wild and cultivated rice in response to the pathogen *Magnaporthe oryzae*. Rice..

[42] Campos-Soriano L, Gómez-Ariza J, Bonfante P, Segundo BS (2011). A rice calcium-dependent protein kinase is expressed in cortical root cells during the pre-symbiotic phase of the arbuscular mycorrhizal symbiosis. BMC Plant Biol.

[43] Virdi AS, Singh S, Pingh P (2015). Abiotic stress responses in plants: roles of calmodulin-regulated proteins. Front Plant Sci.

[44] Hu R, Wang Z, Wu P, Tang J, Hou X (2015). Identification and abiotic stress analysis of calmodulin-binding transcription activator/signal responsive genes in non-heading Chinese cabbage (*Brassica campestris* ssp. *chinensis* Makino). Plant Omics.

[45] Meng X, Zhang S (2013). MAPK cascades in plant disease resistance signaling. Annu Rev Phytopathol.

[46] Huang D, Ma M, Wang Q, Zhang M, Jing G, Li C (2020). Arbuscular mycorrhizal fungi enhanced drought resistance in apple by regulating genes in the mapk pathway. Plant Physiol Biochem.

[47] Li Y, Liu Z, Hou H, Lei H, Zhu X, Li X (2013). Arbuscular mycorrhizal fungi-enhanced resistance against *Phytophthora sojae* infection on soybean leaves is mediated by a network involving hydrogen peroxide, jasmonic acid, and the metabolism of carbon and nitrogen. Acta Physiol Plant.

[48] Jiang JJ, Ma SH, Ye NH, Jiang M, Cao JS, Zhang J (2017). WRKY transcription factors in plant responses to stresses. J Integ Plant Biol.

[49] Wang L, Gao XQ, Zhu LH, Zhou YL, Li ZK (2011). Advances in research on function of WRKY transcription factor genes in plant resistance. J Plant Genet Resour.

[50] Wang K, Li C, Lei C, Zou Y, Fang Y (2021). Dual function of VvWRKY18 transcription factor in the β-aminobutyric acid-activated priming defense in grapes. Physiol Plantarum.

[51] Yang J, Wang Q, Luo H, He C, An B (2020). HbWRKY40 plays an important role in the regulation of pathogen resistance in hevea brasiliensis. Plant Cell Rep.

[52] Wang GS. Studies on fungal community in replanted soil around Bohai gulf and alleviation abble replanted disease by mixed cropping with *album fistulosum* L. Doctoral dissertation. 2019; https://doi.org/CNKI:CDMD:1.1019.013816.

[53] Giovannetti M, Mosse B (1980). An evaluation of techniques for measuringvesicular-arbuscular mycorrhizae in roots. New Phytol.

[54] Parkhomchuk D, Borodina T, Amstislavskiy V, Banaru M, Hallen L, Krobitsch S (2009). Transcriptome analysis by strand-specific sequencing of complementary DNA. Nucleic Acids Res.

[55] Liao Y, Smyth GK, Shi W (2014). featureCounts: an efficient general purpose program for assigning sequence reads to genomicfeatures. Bioinformatics.

[56] Love MI, Huber W, Anders S (2014). Moderated estimation of fold change and dispersion for RNA-seq data with DESeq2. Genome Biol.

[57] Goldstein LD, Cao Y, Pau G, Lawrence M, Wu TD, Seshagiri S (2016). Prediction and quantification of splice events from RNA-Seq data. PLoS One.

[58] Thimm O, Blasing O, Gibon Y, Nagel A, Meyer S, Kruger P (2004). MAPMAN: a user-driven tool to display genomics data sets onto diagrams of metabolic pathways and other biological processes. Plant J.

[59] Zhang J, Xu HF, Wang N, Jiang SH, Fang HC, Zhang ZY (2018). The ethylene response factor MdERF1B regulates anthocyanin and proanthocyanidin biosynthesis in apple. Plant Mol Biol.

