# Supplementary figures and images for: Involvement of MdWRKY40 in the defense of mycorrhizal apple against fusarium solani
Source: BMC Plant Biol. 2022 Aug 2;22:385. doi: 10.1186/s12870-022-03753-z (PMC9344649; doi:10.1186/s12870-022-03753-z)

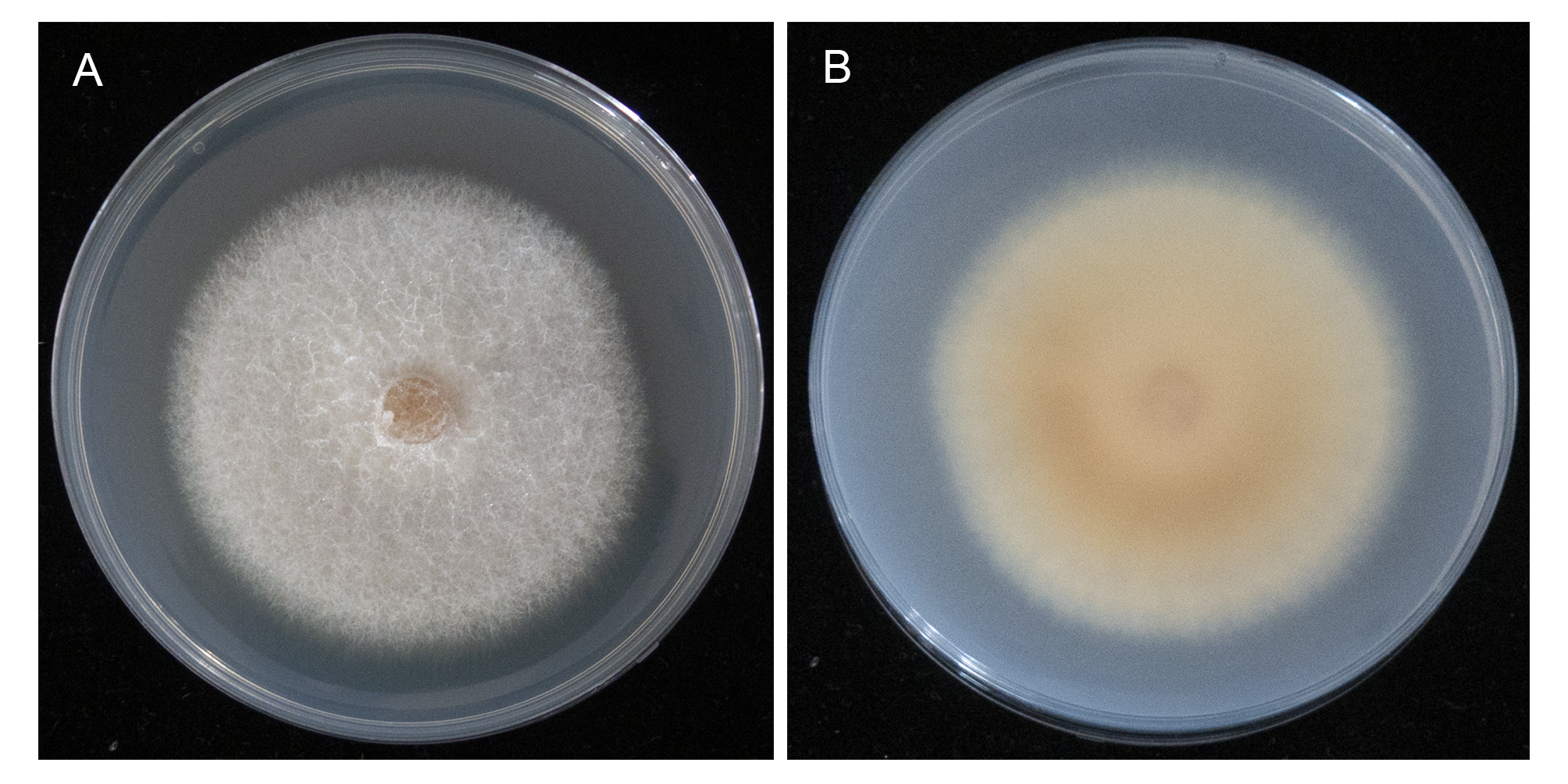

Supplement: Supplementary file 4 — Additional file 4: Supplementary Fig. S1. The morphological observation of F. solani. [file 12870_2022_3753_MOESM4_ESM.tif]

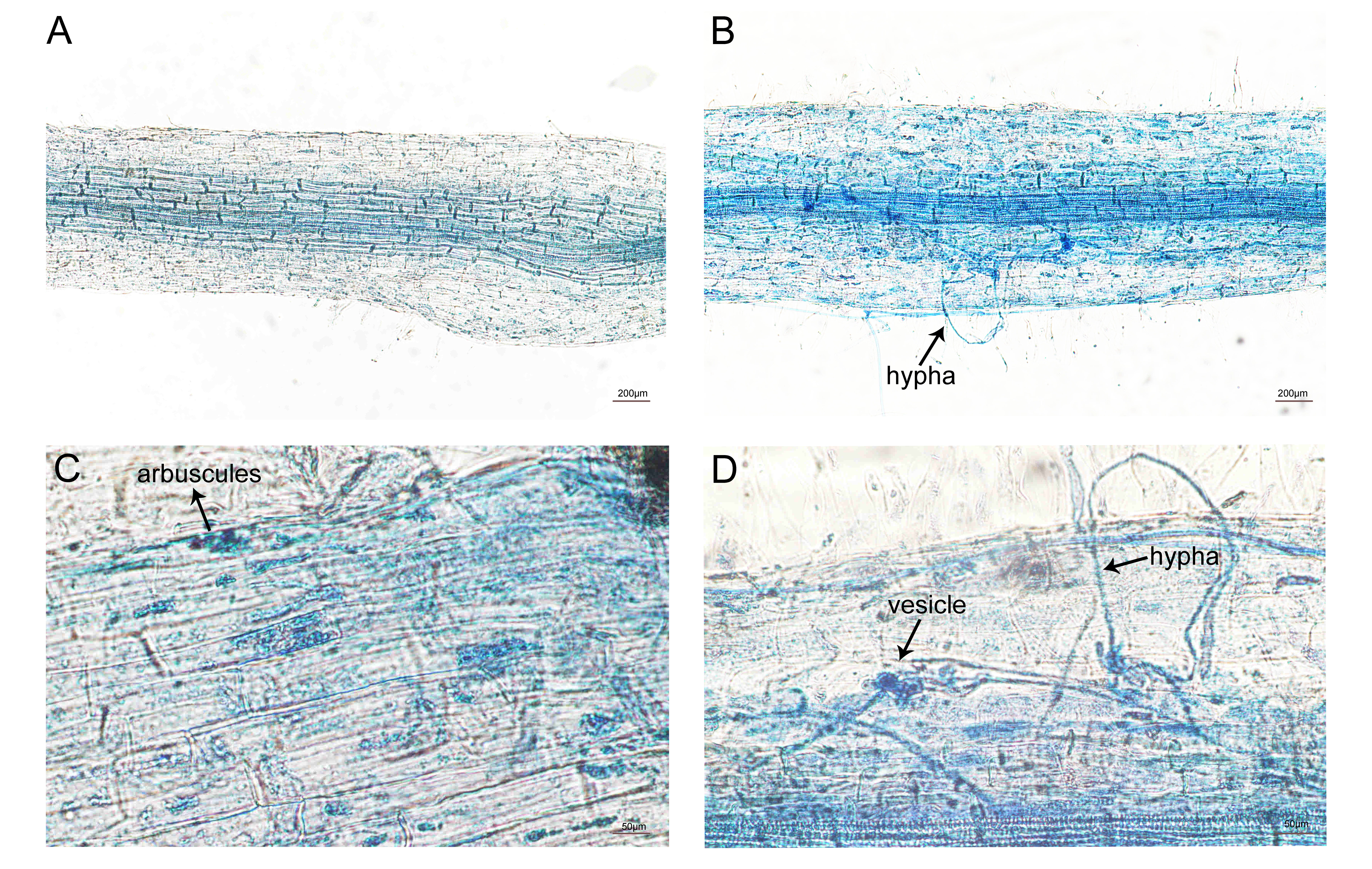

Supplement: Supplementary file 5 — Additional file 5: Supplementary Fig. S2. The development of Paraglomus sp. SW1 in M9T337 seedling roots. [file 12870_2022_3753_MOESM5_ESM.tif]

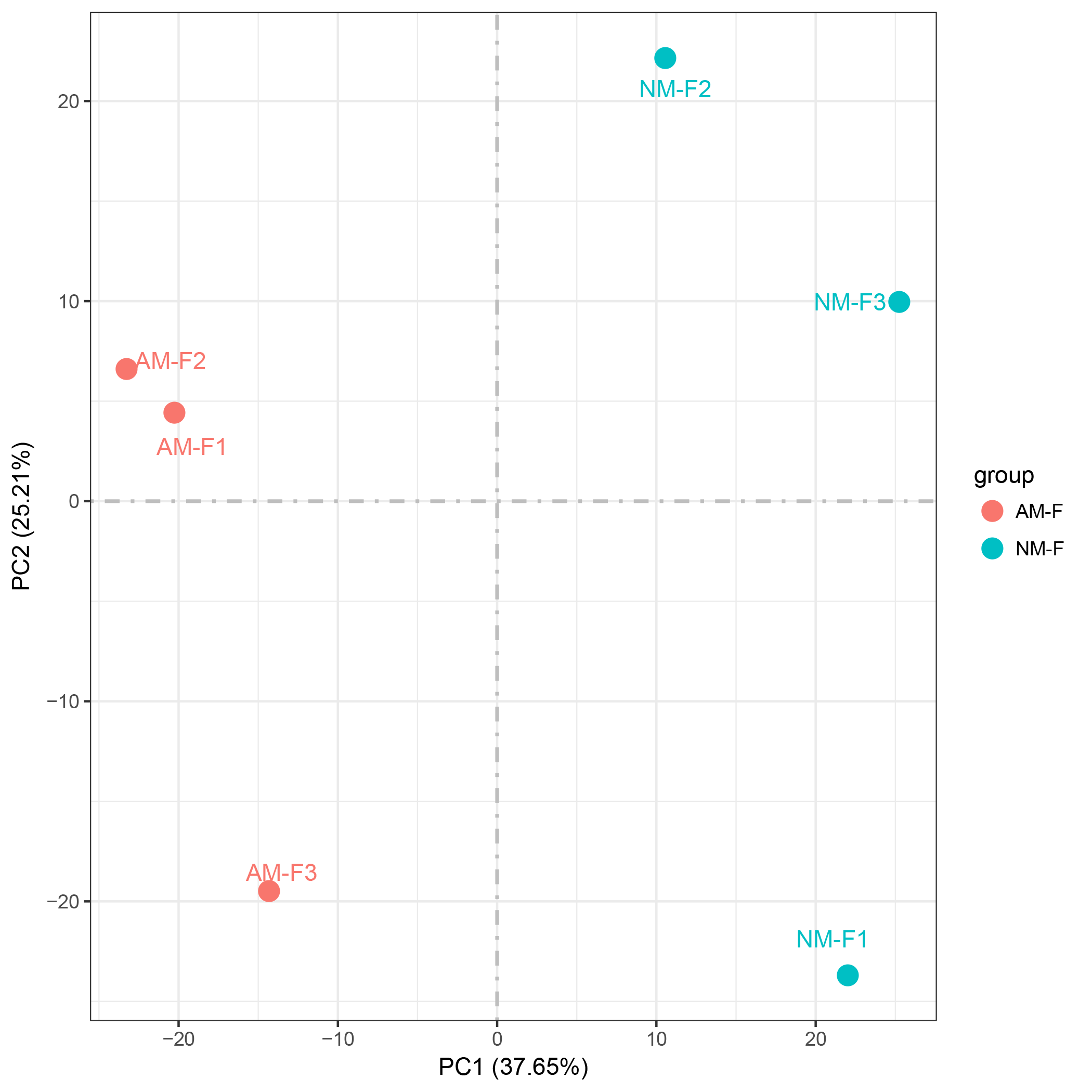

Supplement: Supplementary file 6 — Additional file 6: Supplementary Fig. S3. Principal Coordinate Analysis of the transcriptome. [file 12870_2022_3753_MOESM6_ESM.tif]

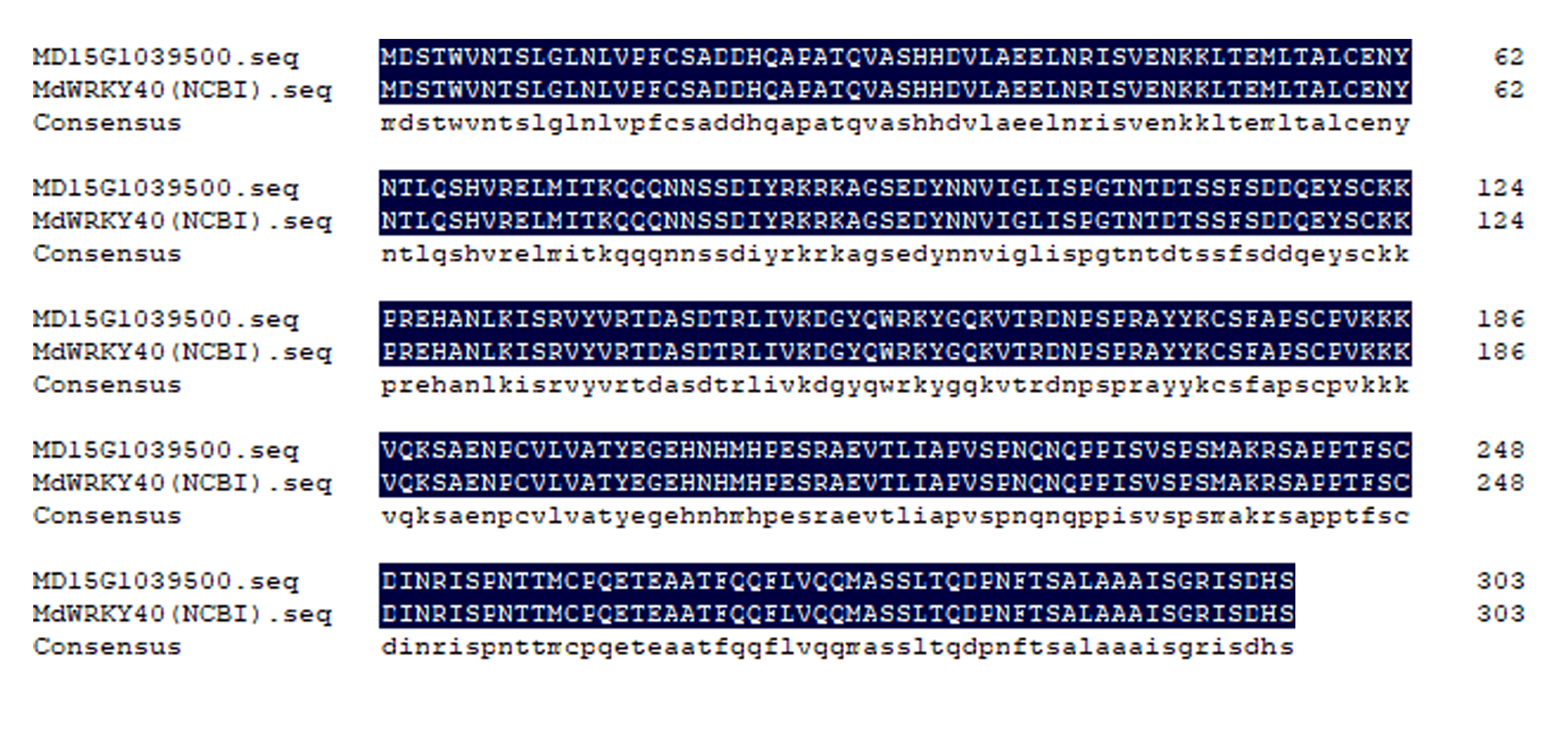

Supplement: Supplementary file 7 — Additional file 7: Supplementary Fig. S4. MdWRKY40 and MD15G1039500 sequence alignment. [file 12870_2022_3753_MOESM7_ESM.tif]

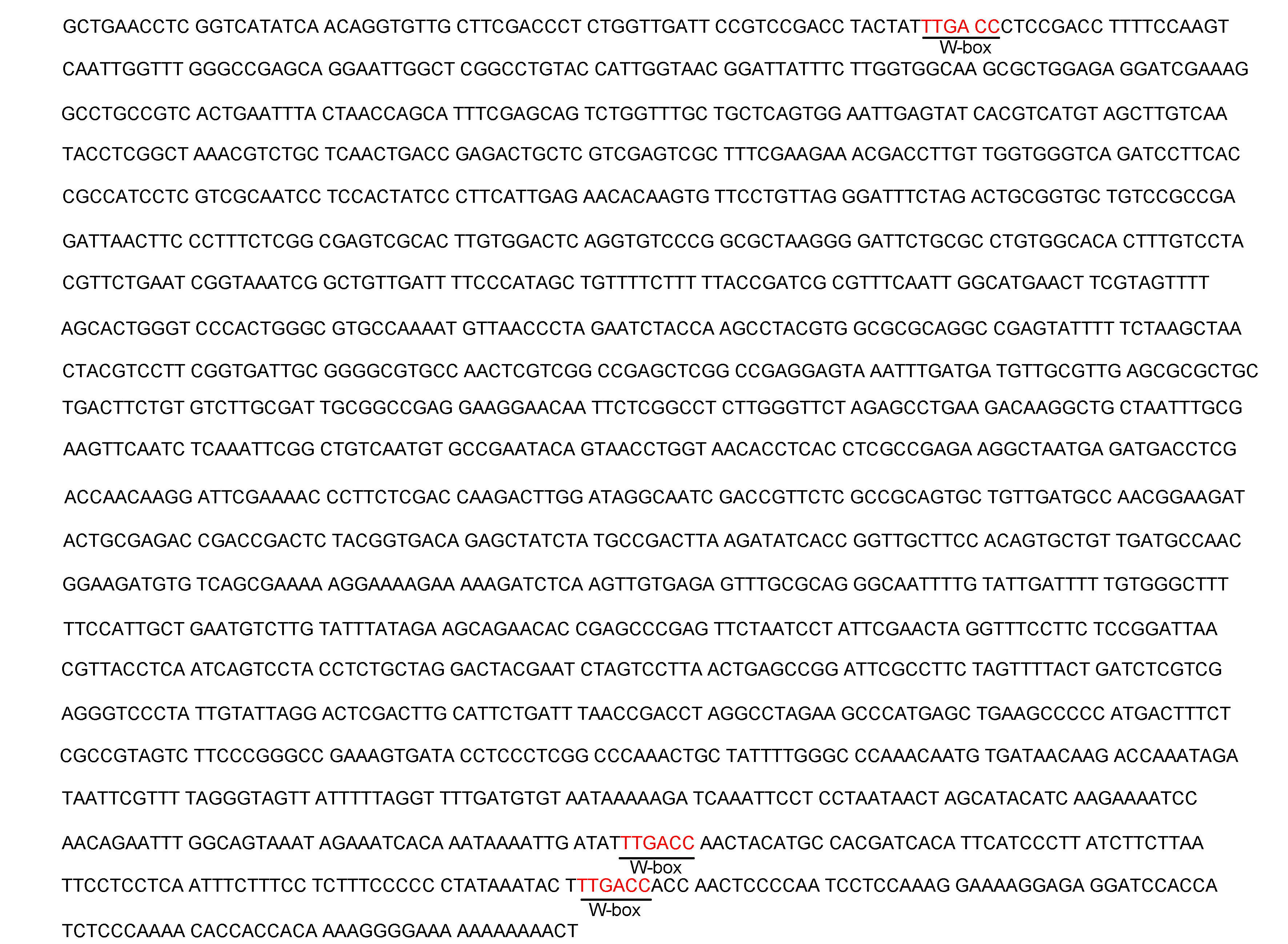

Supplement: Supplementary file 8 — Additional file 8: Supplementary Fig. S5. Analysis of the MdGLU promotor sequence. [file 12870_2022_3753_MOESM8_ESM.tif]

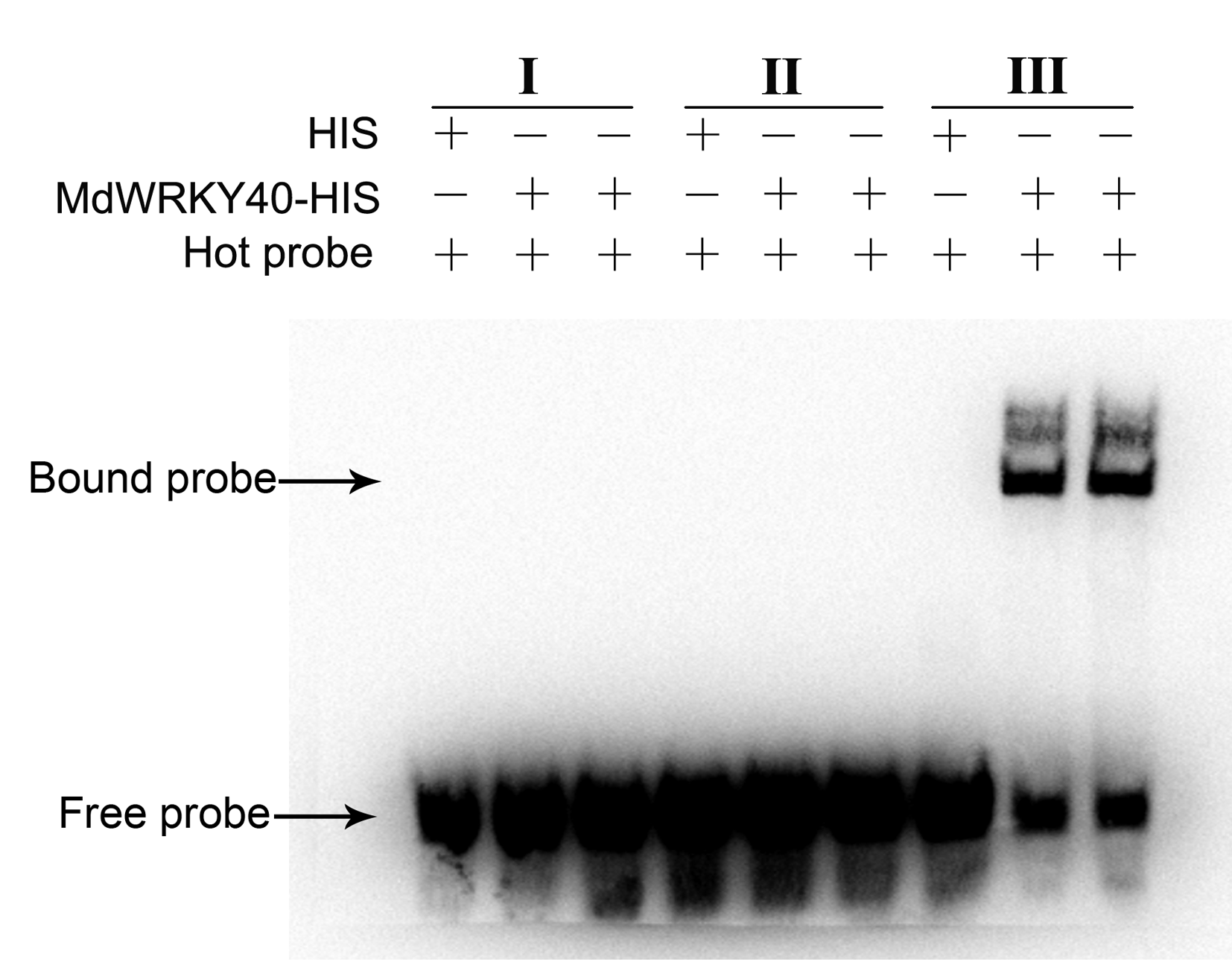

Supplement: Supplementary file 9 — Additional file 9: Supplementary Fig. S6. Electrophoretic mobility shift assay (EMSA) showing the binding of MdWRKY40 to the W-box motif in the promoters of MdGLU. [file 12870_2022_3753_MOESM9_ESM.tif]

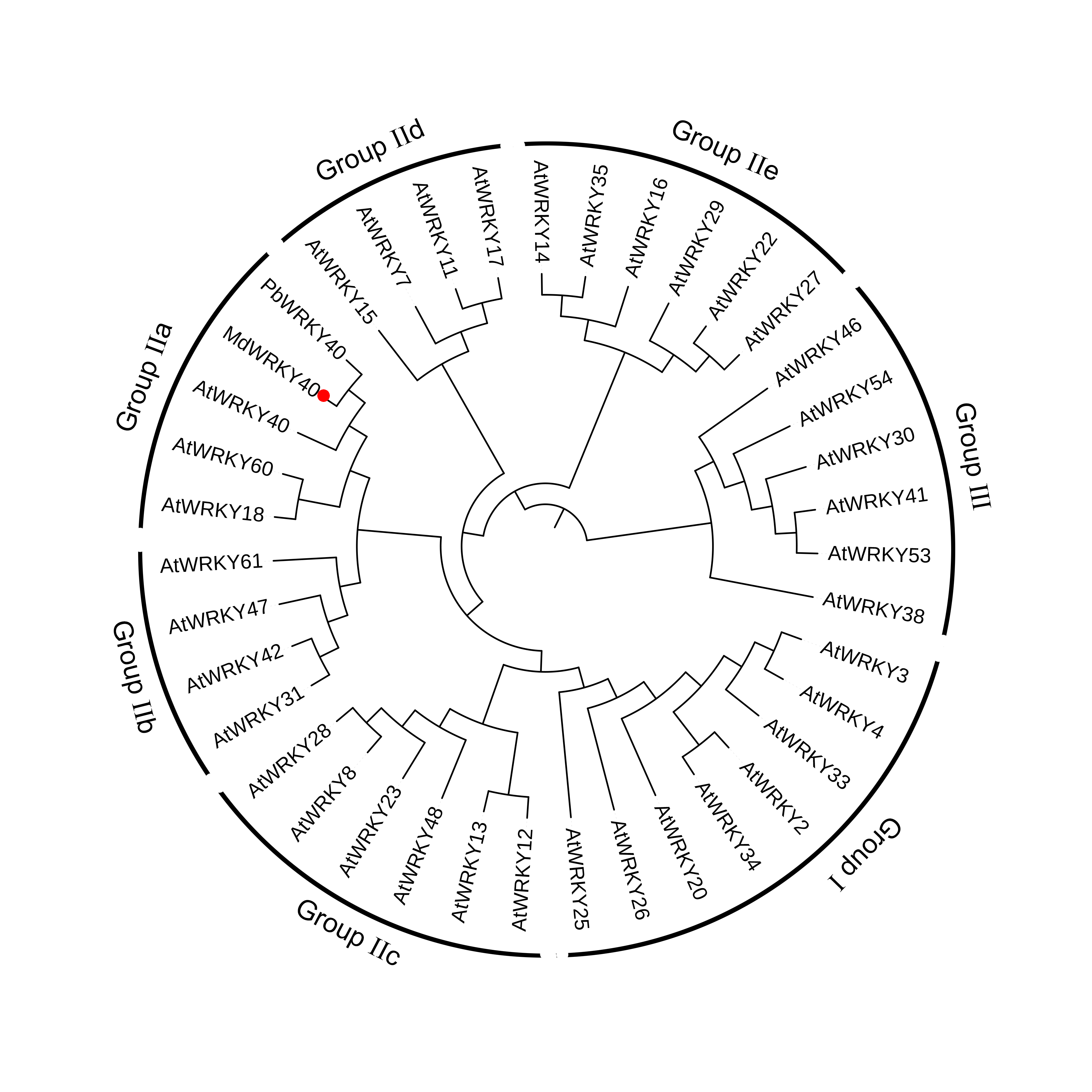

Supplement: Supplementary file 10 — Additional file 10: Supplementary Fig. S7. Phylogenetic tree constructed from protein sequences for WRKY transcription factors. The Arabidopsis thaliana WRKYs were obtained from the TAIR database (https://www.arabidopsis.org/). [file 12870_2022_3753_MOESM10_ESM.tif]
